# A computational framework to establish data-driven constitutive models for time- or path-dependent heterogeneous solids

**DOI:** 10.1038/s41598-021-94957-0

**Published:** 2021-08-05

**Authors:** Weijian Ge, Vito L. Tagarielli

**Affiliations:** Department of Aeronautical Engineering, Imperial College London, Imperial College Road, London, SW7 2AZ UK

**Keywords:** Theory and computation, Computational methods

## Abstract

We propose and implement a computational procedure to establish data-driven surrogate constitutive models for heterogeneous materials. We study the multiaxial response of non-linear n-phase composites via Finite Element (FE) simulations and computational homogenisation. Pseudo-random, multiaxial, non-proportional histories of macroscopic strain are imposed on volume elements of n-phase composites, subject to periodic boundary conditions, and the corresponding histories of macroscopic stresses and plastically dissipated energy are recorded. The recorded data is used to train surrogate, phenomenological constitutive models based on neural networks (NNs), and the accuracy of these models is assessed and discussed. We analyse heterogeneous composites with hyperelastic, viscoelastic or elastic–plastic local constitutive descriptions. In each of these three cases, we propose and assess optimal choices of inputs and outputs for the surrogate models and strategies for their training. We find that the proposed computational procedure can capture accurately and effectively the response of non-linear n-phase composites subject to arbitrary mechanical loading.

## Introduction

All solids display a heterogeneous microstructure and their mechanical response depends on complex mechanisms spanning multiple length- and time-scales. For this reason, recent research has focused on multi-scale computational modelling of different types of materials^1,2^. This is typically conducted via computational homogenization, commonly of the 1st order^3^, across multiple scales^1,3,4^. Such multiscale calculations often have prohibitive computational cost, such that several researchers have been working at reduced-order modelling (ROM) and surrogate modelling, which is the subject of the current investigation.

In ROM, the original problem is mapped to a lower-dimensional problem space, saving computational cost but reducing the fidelity of the physical description of the material. This mapping occurs by a data-driven method and can be applied to the system equations (as in the R3M method^5^), to the spatial fields of quantities that are being sought^2,6–9^, or to the information describing the microstructure^10^. Reduced-order models typically have neglected microstructure evolution, which limits their predictive capabilities in some nonlinear problems.

In surrogate modelling the predictions of detailed and computationally demanding physically-based models are used to perform regressions, using for example Neural Networks (NNs)^11^ and Gaussian processes^12^, also in the case of history-dependent responses^13^. Some of these models capture low-dimensional representations of the field of variables of interest through data compression^14^; others use physical governing laws to achieve higher accuracy and to accelerate the training process^15^. Some authors have suggested developing ‘data-driven mechanics’^16,17^ informed exclusively by detailed measurements.

In the current paper, we focus on using NNs to develop surrogate constitutive models for heterogeneous solids. This is of interest for two reasons: first, because it enables computational savings of multiple order of magnitudes when performing multiscale simulations; secondly, it poses the theoretical basis for the development of surrogate models obtained directly from measured data. The existing literature has not explored quantitatively how the heterogeneity of the solid under investigation affects the predictions of a surrogate model, and for this reason, we focus on the response of a general n-phase composite with tuneable heterogeneity. While many of the existing approaches rely on assuming, to some extent, the mathematical structure of the surrogate model (for example the existence of a yield surface, or the existence of an associated flow rule), we aim at constructing surrogate models that are purely data-driven. Most studies have focused on the response of solids to monotonic proportional loading, while here we aim at achieving generality of the loading, such as to obtain surrogate models that can be employed effectively in cases where the loading is far from proportional, for example in impact applications.

We study separately the cases of nonlinear elastic, time-dependent and history-dependent local constitutive response (hyperelastic, viscoelastic or elastoplastic, respectively), and we identify optimal modelling strategies, including the optimal features of the NNs, for each case, and accompanying procedures for the training of the models. The computational framework is presented in “Computational framework” while results are shown and discussed in “Results and discussion”.

## Computational framework

In this study we analyse volume elements (VEs) of n-phase composites. We simulate their mechanical response using FE calculations and computational homogenisation, and we employ the results of such calculations to assemble a training dataset used to teach the *macroscopic* material behaviour to neural networks. The choice of using neural networks over other machine learning techniques is driven by the ready availability of computational tools, but many of the conclusions of the present study can be extended to other machine learning methods. In this section, we describe the steps of the process.

### Definition of the VEs

**Figure 1:**
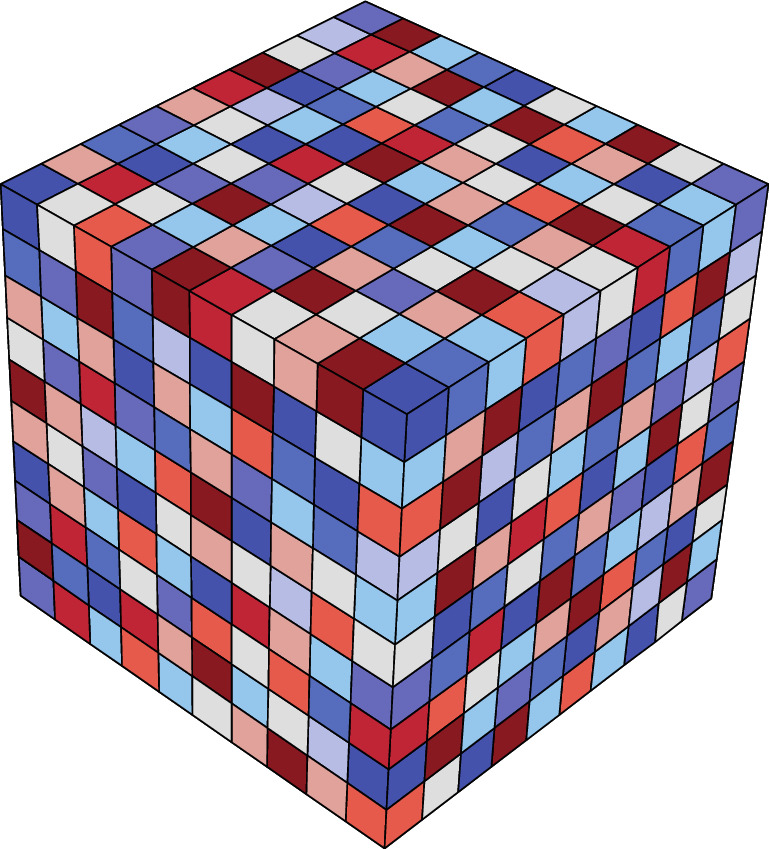
A realisation of the n-phase composite investigated in this study.

In this study we simulate the response of model heterogeneous materials; these consist of a cubic domain of unit volume discretised into $10 \times 10 \times 10$ cubic cells, each possessing different mechanical properties, as sketched in Fig. 1. Each of such cells is meshed by one single eight-noded finite element of type C3D8 in Abaqus^18^. The choice of the density of the discretisation was made to ensure that the VEs displayed a reasonably complex and approximately isotropic macroscopic mechanical response while keeping the computational cost low. This model material is not intended to quantitatively represent any real solid, but rather to represent n n-phase composite of easily tuneable heterogeneity; the spatial distribution $P\left( {x_{i} } \right)$ of a mechanical property $P$ of the composite under investigation is assumed to follow

1 $$P\left( {x_{i} } \right) = P_{\min } + r_{i} \left( {P_{\max } - P_{\min } } \right)$$

where $r_{i} \in \left[ {0,1} \right]$ represents random numbers of uniform probability density generated at the position of each cell, $x_{i}$, $i = 1,2, \ldots 1000$. We quantify the randomness of each mechanical property $P$ by its relative variance, defined as

2 $$\kappa = 2\left( {P_{\max } - P_{\min } } \right)/\left( {P_{\max } + P_{\min } } \right) \in \left[ {0,2} \right]$$

where $\kappa = 0$ corresponds to no spatial variation of *P* and $\kappa = 2$ corresponds to the maximum variation, obtained in the case $P_{\min } = 0$ (in which it is also $P_{\max } = 2\overline{P}$, where $\overline{P}$ is the average value of *P*). The choice of an uncorrelated, uniform probability density for the mechanical properties of each phase of the composite was based on the ease of implementation. In the course of this study, we will analyse the performance of only one realization of the VEs for each of the three types of constitutive models analysed.

### Finite element simulations

FE simulations were conducted in Abaqus Standard^18^ and were fully automated via Python scripts. Periodic boundary conditions were prescribed on the three pairs of opposite faces of the VEs, as prescribed in^19^. The macroscopic true strains on the VE were controlled by prescribing appropriate displacements on a set of auxiliary nodes; such displacements controlled the distance between opposite faces of the cuboidal domains and also allowed to calculate the current area of all the faces of the VE. The corresponding reaction forces at these auxiliary nodes, representing the components of the total forces acting on the faces of the VE, were recorded during the simulations and used, together with the current area of the faces, to calculate the histories of macroscopic true stress components, as described in detail in^3,19^.

In this study, we analyse VEs that are initially approximately isotropic and are subject to small strains, such that strain-induced anisotropy is negligible. Driven by the need to keep the computational cost low, we assume that the material response can be evaluated in principal strain space, which coincides with principal stress space for our isotropic material. In the FE simulations, we prescribe time histories of only the normal macroscopic strains $\varepsilon_{xx} ,\,\varepsilon_{yy} ,\varepsilon_{zz}$, while the macroscopic shear strains are set to zero, $\varepsilon_{xy} ,\,\varepsilon_{xz} ,\varepsilon_{yz} = 0$ (*xyz* is a reference system aligned with the edges of the cubic VE). In other words, the applied strains $\varepsilon_{xx} ,\,\varepsilon_{yy} ,\varepsilon_{zz}$ are interpreted as principal strains $\varepsilon_{I} ,\,\varepsilon_{II} ,\varepsilon_{III}$. We check that the macroscopic shear tractions are negligibly small in the simulations, $\tau_{xy} ,\,\tau_{xz} ,\tau_{yz} \approx 0$, confirming that the global reference system *xyz* is a principal system also for the macroscopic stress tensor. We analyse separately three local constitutive behaviours, using models readily available in Abaqus. We summarise these models briefly here.

#### Neohookean hyperelastic

A hyperelastic composite was studied as a reference case. For this conservative material, a one-to-one correspondence exists between stress and strain tensors, and we expect a NN to comfortably capture such correspondence. Due to the small deformation imposed, the response of such hyperelastic model practically coincides with that of a linear elastic model of equal initial elastic constants. Therefore, the effects of elastic nonlinearity in the structural response are not examined in this study. We model an array of isotropic, hyperelastic cubic cells, each having an initial Poisson’s ratio of 0.45 and an initial Young’s modulus randomly assigned as in Eq. (1) and having a spatial average of 3 GPa. A time-independent neohookean local strain energy density is assumed.

#### Linear viscoelastic

We also consider a model where the local response is taken as linear isotropic viscoelastic. Such response is defined in Abaqus in terms of instantaneous Young’s modulus and Poisson’s ratio and of the time-decay of the shear and bulk moduli, expressed by the Prony series^20^. In this study the Poisson’s ratio is taken as constant in time and space and equal to 0.45, while the instantaneous Young’s modulus is randomly assigned according to Eq. (1) and has an average value of 3 GPa. The time decay law is taken as spatially uniform across the VE and two different laws are considered: one consisting of a single-term Prony series, and one consisting of three terms of such series, each associated with time decay constants of different orders of magnitude. A summary of the relevant properties for the viscoelastic composites is provided in Table 1.

**Table 1:** Relevant properties of the viscoelastic composites.

Viscoelastic with 1 term of Prony series		
Instantaneous elastic constants	$\overline{E}_{0}$ = 3Gpa	$\nu_{0}$ = 0.45
Prony coefficients	$\mu_{1} = 0.5, \, k_{1} = 0.5, \, t_{1} = 1\,\,{\text{s}}\,$	

Viscoelastic with 3 terms of Prony series		
Instantaneous elastic constants	$\overline{E}_{0}$ = 3Gpa	$\nu_{0}$ = 0.45
Prony coefficients	$\mu_{1} = 0.177, \, k_{1} = 0.177, \, t_{1} = 7.71\,\,10^{ - 3} \,{\text{s}}$ $\mu_{2} = 0.074, \, k_{2} = 0.074, \, t_{2} = 0.21\,{\text{s}}$ $\mu_{3} = 0.017, \, k_{3} = 0.017, \, t_{3} = 3.88\,{\text{s}}$	

#### Elastic–plastic

Elastic–plastic model composites were assembled by assigning to each cell a linear elastic response and J2 incompressible, rate-independent plasticity with isotropic hardening. We assume that the material has substantial strain hardening, with a constant true hardening modulus of 1 GPa, such that the local material response is defined by two elastic constants, a yield stress and this hardening modulus, which is assumed to be uniform. The Poisson’s ratio is also assumed to be spatially uniform and equal to 0.45, while spatial variations of Young’s modulus and the yield stress are considered separately and prescribed according to Eq. (1), using average values of 3 GPa for the elastic modulus and 50 MPa for the yield stress.

The FE analyses were conducted via Abaqus Standard using adaptive time stepping. A ‘general step’ was used for the hyperelastic and elastic–plastic models, while a ‘visco step’ was used for the viscoelastic model. In all simulations, we accounted for geometric non-linearity.

### Generation of the training datasets

To generate the training datasets we consider, for each type of constitutive model, a single realisation of the VE described in “Definition of the VEs” and “Finite element simulations”. Note that since we analyse only one realisation of the VE, this does not need to be sufficiently large to be “representative”; the only requirement is that such domain is sufficiently large to be approximately isotropic, since we will work in principal stress/strain space. A preliminary RVE size convergence analysis showed that the VEs analysed had an intrinsic scatter of approximately 5%, and that a further increase in the number of statistical cells would not affect the average response considerably (while it would reduce such intrinsic scatter). With regards to the FE discretisation, the response of the domain is mildly sensitive to the mesh; however, considering domains with a finer FE mesh merely results in a marginally different (weaker) macroscopic material response.

**Figure 2:**
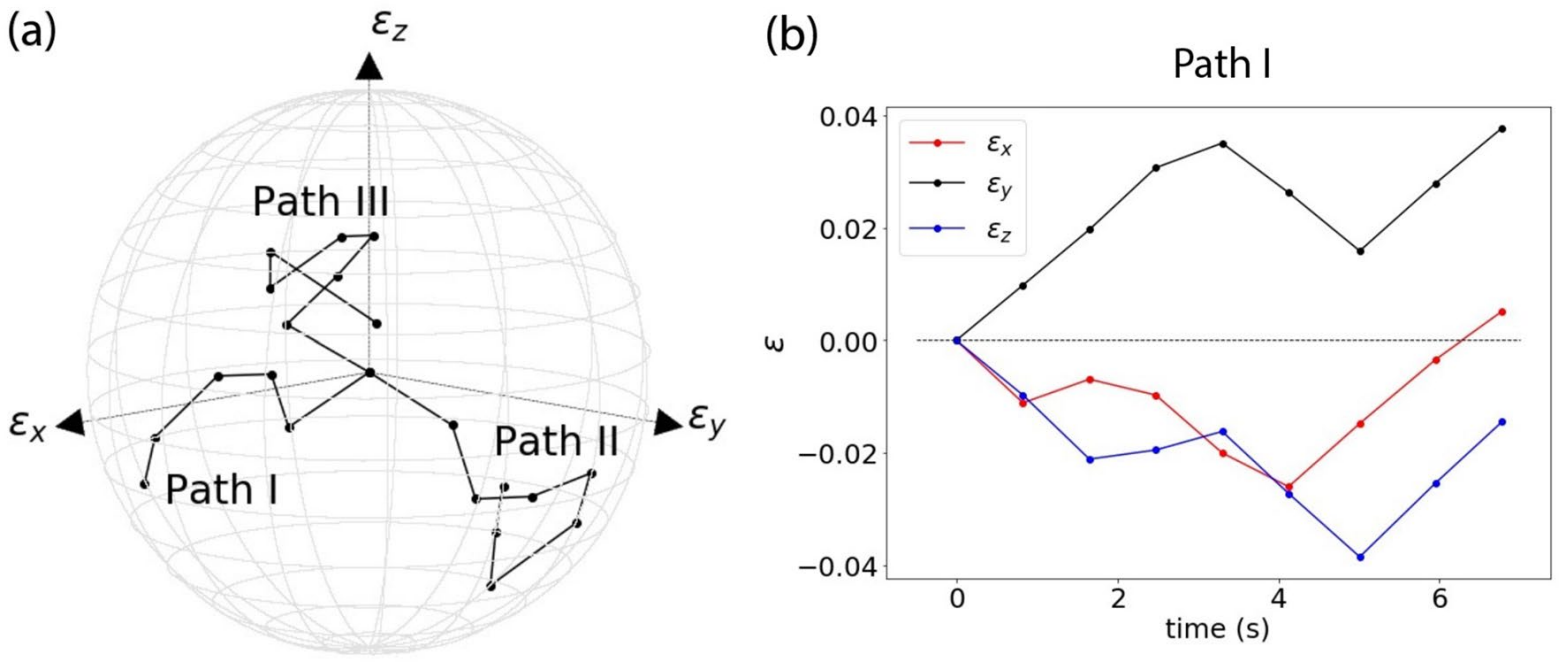
Illustration of the random strain histories imposed on the VEs to generate the training data. (a) Three examples of pseudo-random loading paths. (b) Details of the strain histories associated to Path 1.

Displacement boundary conditions are applied to the auxiliary nodes of the VE to prescribe appropriate histories of strain and strain rate. The VEs are taken for pseudo-random walks in true principal strain space $\left( {\varepsilon_{xx} ,\,\varepsilon_{yy} ,\varepsilon_{zz} } \right).$ The random walks comprise a number of consecutive steps of random amplitude and duration; each random walk is terminated upon meeting the condition $\sqrt {\varepsilon_{xx}^{2} + \,\varepsilon_{yy}^{2} + \varepsilon_{zz}^{2} } \ge \varepsilon_{\max } = 0.05$, to limit the magnitude of the applied strains, as illustrated in Fig. 2. Each step in the random walks corresponds to relative strain amplitudes (final strain minus initial strain) taken, in the case of the hyperelastic and elastic–plastic models, as

3 $$\Delta \varepsilon_{i} = {\text{sgn}} (r_{{\beta_{i} }} - \theta )[\Delta \varepsilon_{\min } + r_{{\alpha_{i} }} \cdot (\Delta \varepsilon_{\max } - \Delta \varepsilon_{\min } )],\,\,\,\,i = x,y,z$$

4 $${\text{sgn}} (x) = \left\{ {\begin{array}{*{20}ll} { - 1 } & {x \le 0} \\ {1 } & { x > 0} \\ \end{array} } \right.$$

where $\Delta \varepsilon_{\min } = 0.01$ and $\Delta \varepsilon_{\max } = 0.015$ are the minimum and maximum amplitude of the strain step in each Cartesian direction, $r_{{\alpha_{i} }}$ and $r_{{\beta_{i} }}$ are uniform random variables between 0 and 1, and $\theta = 0.5$ defines the probability that the amplitude of each strain step is negative (equal probability of positive and negative steps is assumed in this study).

For rate-independent materials (hyperelastic and elastic–plastic constitutive models), each step in the random walks has a time duration $\Delta T = 1.$ For the rate-dependent viscoelastic material, a different approach is taken: for each step of the pseudo-random walks, three strain rates are randomly generated for each of the Cartesian directions, such that

5 $$\log \dot{\varepsilon } = \eta_{\min } + r_{\gamma } \cdot (\eta_{\max } - \eta_{\min } );\,\,\,\eta_{\min } = \log \left( {\dot{\varepsilon }_{\min } } \right);\,\,\,\eta_{\max } = \log \left( {\dot{\varepsilon }_{\max } } \right)$$

where $r_{\gamma }$ is a uniform random number between 0 and 1 and $\dot{\varepsilon }_{\min } ,\,\dot{\varepsilon }_{\max }$ are the smallest and largest possible strain rates. This ensures that the logarithms of such strain rates are uniformly distributed. Then, the step size $\Delta \varepsilon$ is generated according to Eq. (3) and the time step duration is determined as $\Delta T = \Delta \varepsilon /\max \left( {\dot{\varepsilon }_{x} ,\dot{\varepsilon }_{y} ,\dot{\varepsilon }_{z} } \right)$, where $\dot{\varepsilon }_{x} ,\dot{\varepsilon }_{y} ,\dot{\varepsilon }_{z}$ are the three randomly generated strain rates for each current step.

The FE simulations are then conducted for as many random walks as needed (100 in this study); their solutions are incremental, due to the non-linearity of the problems, and the solution time increments $\Delta t$ are determined adaptively by the solver. We post-process the results to extract the histories of macroscopic true strain and true stress as well as of the plastically dissipated energy $W_{pl}$. We then create a database in which each row represents a general increment of the solution, and the columns report: the time increment $\Delta t$; the initial strains $\varepsilon_{xx}^{i} ,\,\varepsilon_{yy}^{i} ,\varepsilon_{zz}^{i}$; the initial stresses $\sigma_{xx}^{i} ,\,\sigma_{yy}^{i} ,\sigma_{zz}^{i}$; strain increments $\Delta \varepsilon_{xx} = \varepsilon_{xx}^{f} - \varepsilon_{xx}^{i}$, $\Delta \varepsilon_{yy} = \varepsilon_{yy}^{f} - \varepsilon_{yy}^{i}$, $\Delta \varepsilon_{zz} = \varepsilon_{zz}^{f} - \varepsilon_{zz}^{i}$; stress increments $\Delta \sigma_{xx} = \sigma_{xx}^{f} - \sigma_{xx}^{i}$, $\Delta \sigma_{yy} = \sigma_{yy}^{f} - \sigma_{yy}^{i}$, $\Delta \sigma_{zz} = \sigma_{zz}^{f} - \sigma_{zz}^{i}$; increment in plastically dissipated energy, $\Delta W_{pl} = W_{pl}^{f} - W_{pl}^{i}$, where the superscripts *i* and *f* denote values at the beginning (initial) and the end (final) of each increment, respectively. Additional quantities, such as strain rates, macroscopic elastic and plastic strains, equivalent plastic strain are calculated from this data as will be detailed below. All quantities with dimensions of stress are divided by the average Young’s modulus (3 GPa), while the plastically dissipated energy is divided by the average Young’s modulus and by the volume of the VE (equal to 1 in all cases), to obtain a dataset with non-dimensional entries. Each row of such a database represents a data point used to train the surrogate models.

The method proposed to generate the training dataset was the result of a preliminary study in which we assessed the distribution of the generated data over the input space of the surrogate models; it was found that the choices in Eqs. (3)–(5) correspond to an adequate spread of such data, as shown in detail in Appendix A (Supplementary Material). By adjusting the parameters $\varepsilon_{\max }$,$\theta$, $\Delta \varepsilon_{\max }$, $\Delta \varepsilon_{\min }$, $\eta_{\max }$, $\eta_{\min } ,$ the distribution of the training data over the input space can be modified to optimise it for different physical situations. A study of the sensitivity of the results to the above parameters is not pursued here, but rather we employ the parameters described above for all types of models investigated in this study.

A workstation with 2 processors (Intel Xeon e5-2650 v4, 2.20 GHz) and 256 GB of RAM was used in all computations. Approximately 10 h of CPU time were necessary to produce the training dataset.

### Training of the neural networks

We first randomly choose a number $\phi$ of data points from the database described above. A number $\bar{\phi } = 0.9\phi$ of these data points are used to train the NNs, while the remaining $0.1\phi$ points are used for validation. All the coordinates of the training data points are normalised by subtracting their average value and dividing by their variances (averages and variances are calculated over the set of $\bar{\phi }$ training data points). In this study we use multi-layer feed-forward neural networks^21^, with two hidden layers of 100 neurons in all cases; the choice of using the same NN architecture in all cases allows a fair comparison between the performances of the different models produced in this study. Such networks establish a non-linear function relating the desired inputs and outputs. If ${\mathbf{x}}$ and ${\mathbf{y}}$ are column vectors containing the inputs and outputs, respectively, such non-linear function reads

6 $${\mathbf{y = }}NN\left( {\mathbf{x}} \right){\mathbf{ = W}}_{{\mathbf{2}}} f\left( {{\mathbf{W}}_{{\mathbf{1}}} \left( {f\left( {{\mathbf{W}}_{{\mathbf{0}}} {\mathbf{x + b}}_{{\mathbf{0}}} } \right)} \right){\mathbf{ + b}}_{{\mathbf{1}}} } \right){\mathbf{ + b}}_{{\mathbf{2}}}$$

where $f$ is a non-linear activation function, taken in this study as the Rectified Linear (ReLu) function, defined as

7 $$f(\alpha ) = \max (0,\alpha ),$$

${\mathbf{W}}_{0} ,\,{\mathbf{W}}_{{\mathbf{1}}} ,\,{\mathbf{W}}_{2}$ are weight matrices and ${\mathbf{b}}_{0} ,\,{\mathbf{b}}_{{\mathbf{1}}} ,\,{\mathbf{b}}_{2}$ are column vectors of biases. Training of the neural network is a stochastic optimisation process aimed at minimising a chosen objective (or ‘loss’) function. When NNs are used to perform regressions, in this study we choose the loss function to be the mean absolute error (*MAE*), defined as

8 $$MAE = \frac{1}{{\hat{\phi }}}\sum\limits_{j = 1}^{{\hat{\phi }}} {\sum\limits_{k = 1}^{d} {\left| {y_{k}^{\left( j \right)} - \bar{y}_{k}^{\left( j \right)} } \right|} }$$

where $y_{k}^{\left( j \right)} = NN\left( {{\bar{\mathbf{x}}}^{\left( j \right)} } \right)$ is the output of the NN [Eq. (6)] corresponding to the input vector ${\bar{\mathbf{x}}}^{\left( j \right)}$, $\bar{y}_{k}^{\left( j \right)}$ is the corresponding output taken from the training database, $j = 1,2..\bar{\phi }$ is used to sum over each of the training data points and *d* is the length of the vector ${\mathbf{y}}.$ Minimisation of the loss function is achieved by back-propagation, using the Adam algorithm (^22^, with $\beta_{1} = 0.9$, $\beta_{2} = 0.999$, $\varepsilon = 0$) with no regularisation, a maximum of 3000 epochs, a batch size of 50 and learning rate of 0.001. The choice of these parameters was made based on the result of a preliminary study aimed at maximising accuracy while maintaining a reasonable computational cost.

We will discuss below that for the elastic–plastic models, separate neural networks will be used to perform regressions and classifications, and a different loss function will be used in the case of classification, as described in detail in “Response of an elastic–plastic VE”. Training of a single NN took an average CPU time of 20 min.

### Structure of the neural networks

For each of the three constitutive descriptions investigated (hyperelastic, linear viscoelastic, elastic–plastic), we use NNs as a surrogate model to predict the homogenised constitutive response of the VEs. In each case, the input and output vectors ${\mathbf{x}}$ and ${\mathbf{y}}$ in Eq. (6) contain different quantities; in this section, we present and motivate in detail the choice of inputs and outputs for each surrogate model.

#### Response of a hyperelastic VE

For the case of the hyperelastic analyses, each cell (or finite element) of the VE is assigned a local response chosen as neohookean. In such constitutive description, which does not include energy dissipation, the local stresses $\left[ \sigma \right]$ are obtained by differentiating a scalar elastic potential function as

9 $$\left[ \sigma \right] = \frac{1}{J}\frac{\partial W}{{\partial {\mathbf{F}}}}{\mathbf{F}};\,\,\,\,J = \lambda_{1} \lambda_{2} \lambda_{3}$$

where ${\mathbf{F}}$ is deformation gradient, $\lambda_{1} ,\lambda_{2} ,\lambda_{3}$ are principal stretches, and $W$ is the neohookean elastic potential, defined as

10 $$W = C_{10} (I_{1} - 3) + \frac{1}{{D_{1} }}(J - 1)^{2} ;\,\,\,\,\,I_{1} = \lambda_{1}^{2} + \lambda_{2}^{2} + \lambda_{3}^{2} ;\,\,\,\,\,C_{10} = \frac{\mu }{2}, \, D_{1} = \frac{2}{{K_{{}} }}$$

where $\mu$ and $K$ are the local shear and bulk moduli, respectively, and they are related to the local Young’s modulus $E$ and Poisson’s ratio $\nu$ by the usual relations

11 $$\mu = \frac{E}{2(1 + \nu )}, \, K = \frac{E}{3(1 - 2\nu )}$$

The hyperelastic VEs are n-phase composites in which each phase has a different Young’s modulus and a Poisson’s ratio $\nu = 0.45$; their macroscopic response is conservative. An elastic potential could be introduced to describe such macroscopic response, however, this would take a form different, in general, from the local potential in Eq. (10). Such potential would establish a non-linear one-to-one mapping of the macroscopic true stress and true strain tensors. In our surrogate models, the neural network (6) will establish such correspondence, such that

12 $${\mathbf{x}} = \left( {\varepsilon_{xx} ,\varepsilon_{yy} ,\varepsilon_{zz} } \right)^{T} = {{\varvec{\upvarepsilon}}};\,\,\,\,{\mathbf{y}} = \left( {\sigma_{xx} /\overline{E},\sigma_{yy} /\overline{E},\sigma_{zz} /\overline{E}} \right)^{T} = {{{{{\varvec{\upsigma}}}}}}/\overline{E} = NN\left( {\mathbf{x}} \right)$$

where $\overline{E}$ is the average Young’s modulus of the VE. The choice of having true principal strains ${{\varvec{\upvarepsilon}}}$ as input and principal stresses ${{{{{\varvec{\upsigma}}}}}}$ as an output, rather than vice-versa, is convenient since our simulations are conducted in strain control.

#### Response of a linear viscoelastic VE

In this case, the VEs are assigned linear viscoelastic local constitutive response, as

13 $$\begin{gathered} {{{{{\varvec{\upsigma}}}}}} = {{{{{\varvec{\upsigma}}}}}}^{dev} + {\mathbf{I}}\sigma^{vol} ;\,\,\,\,\,{{{{{\varvec{\upsigma}}}}}}^{dev} = 2\int_{0}^{t} {\mu_{R} (t - t^{\prime})\frac{{\partial {{\varvec{\upvarepsilon}}}^{dev} (t^{\prime})}}{\partial t^{\prime}}dt} ;\,\,\,\,\,\sigma^{vol} = \int_{0}^{t} {K_{R} (t - t^{\prime})\frac{{\partial \varepsilon^{vol} (t^{\prime})}}{\partial t^{\prime}}dt} \hfill \\ \mu_{R} (t)/\mu_{0} = 1 - \sum\limits_{k = 1}^{N} {\mu_{k} \left( {1 - e^{{ - t/t_{k} }} } \right)} ;\,\,\,\,\,K_{R} (t)/K_{0} = 1 - \sum\limits_{k = 1}^{N} {k_{k} \left( {1 - e^{{ - t/t_{k} }} } \right)} ,\,\,\,k_{k} = \mu_{k} . \hfill \\ \end{gathered}$$

where $\mu_{0}$ and $K_{0}$ are the instantaneous shear and bulk moduli, respectively. The principal stress vector ${{{{{\varvec{\upsigma}}}}}}$ is decomposed into its deviatoric and hydrostatic parts as in Eq. (13) (note $${\mathbf{I}} = \left( {\begin{array}{*{20}c} 1 & 1 & 1 \\ \end{array} } \right)^{T}$$), and the shear and bulk moduli $\mu_{R} (t)$ and $K_{R} (t)$ are assumed to relax according to identical Prony series^20^, having $N$ terms.

The macroscopic response of the composite VE can be proven to be also linear viscoelastic^23^. It can also be shown that the change in principal stresses $\Delta {{{{{\varvec{\upsigma}}}}}}$ over a generic time increment $\Delta t$ can be approximated by

14 $$\begin{aligned} \Delta {{{{{\varvec{\upsigma}}}}}} = & {\mathbf{E}}_{0} \bigg[\frac{{\Delta {{\varvec{\upvarepsilon}}}}}{\Delta t} \circ \left( {\Delta t\,{\mathbf{I}} - \sum\limits_{k = 1}^{{\overline{N}}} {\overline{t}_{k} \left( {\frac{\Delta t}{{\overline{t}_{k} }} + e^{{ - \frac{\Delta t}{{\overline{t}_{k} }}}} - 1} \right){{\varvec{\upgamma}}}_{k} } } \right) + \\ & - {{\varvec{\upvarepsilon}}} \circ \sum\limits_{k = 1}^{{\overline{N}}} {\left( {1 - e^{{ - \frac{\Delta t}{{\overline{t}_{k} }}}} } \right)} {{\varvec{\upgamma}}}_{k} + \left( {1 - e^{{ - \frac{\Delta t}{{\overline{t}_{N - 1} }}}} } \right)\left( {{{\varvec{\upvarepsilon}}} - {\mathbf{E}}_{0}^{ - 1} {{{{{\varvec{\upsigma}}}}}}} \right) \bigg] \\ \end{aligned}$$

where ${\mathbf{E}}_{0}$ is the instantaneous stiffness matrix, ${{\varvec{\upgamma}}}_{k}$ is relative relaxation tensor, $\circ$ indicates the Hadamard product, $\overline{N}$ is the number of terms in the Prony series which approximates the macroscopic relaxation response, and $\overline{t}_{k}$ is the time relaxation constant of the *k*-th term of such Prony series.

Inspired by the structure of Eq. (14), in our machine learning exercise we use

15 $${\mathbf{x}} = \left( {{{\varvec{\upvarepsilon}}},\,\,{{{{{\varvec{\upsigma}}}}}}/\overline{E}_{0} ,\,\,t_{\max } {\dot{\mathbf{\varepsilon }}},\,\,\Delta t/t_{\max } } \right)^{T} ;\,\,\,\,\,{\mathbf{y}} = \Delta {{{{{\varvec{\upsigma}}}}}}/\overline{E}_{0} = NN\left( {\mathbf{x}} \right)$$

where $\overline{E}_{0}$ is the average instantaneous Young’s modulus of the VE, $\Delta {{{{{\varvec{\upsigma}}}}}}$ is a column vector of the increments in principal stresses over a time increment $\Delta t$, corresponding to an increment in principal strains $\Delta {{\varvec{\upvarepsilon}}} = {\dot{\mathbf{\varepsilon }}}\,\Delta t$, $t_{\max }$ is the largest of the time decay constants used in the local Prony series described in Eq. (13). The proposed regression neglects the effect of history-dependent viscous strains on the behaviour of the composite, assuming that the history dependence of the response can be quantified by knowledge of the macroscopic stresses and strains.

#### Response of an elastic–plastic VE

For the case of elastic–plastic VEs, the material is assigned a local elastic–plastic response consisting of isotropic linear elasticity and incompressible, rate-independent J2 plasticity with isotropic hardening and strain hardening given by $\dot{\bar{\varepsilon }}^{pl} = \dot{\bar{\sigma }}/H$, where

16 $$\bar{\sigma } = \sqrt {\frac{3}{2}{\mathbf{S}}:{\mathbf{S}}} ;\,\,\,\,\,\bar{\varepsilon }^{pl} = \int\limits_{0}^{t} {\sqrt {\frac{2}{3}{\dot{\mathbf{\varepsilon }}}_{loc}^{pl} {\dot{\mathbf{\varepsilon }}}_{loc}^{pl} } } dt$$

are the von Mises equivalent stress and accumulated equivalent plastic strain, respectively, such that $\sigma_{ij} \dot{\varepsilon }_{ij} = \bar{\sigma }\dot{\bar{\varepsilon }}^{pl}$ (Einstein’s summation convention applies), ${\mathbf{S}}$ is the local deviatoric stress tensor, ${{\varvec{\upvarepsilon}}}_{loc}^{pl}$ is the local plastic strain tensor, and *H* is a constant hardening modulus.

In this study, we will compare the performance of three different surrogate models based on multiple NNs with different features. The formulation of each of these three surrogate models comprises three steps, as follows.

(i) A linear perturbation analysis of the unloaded VE is conducted in Abaqus to determine the initial macroscopic elastic stiffness matrix of the VE. This is achieved by imposing a small macroscopic normal strain on the VE (in turn, along each of the three Cartesian directions) while constraining the normal macroscopic strains in the other two Cartesian directions to vanish, and extracting the corresponding macroscopic stresses in all directions, such to assemble the 3 by 3 stiffness matrix, written in principal stress/strain space. As the study is limited to small strains and isotropic VEs, we assume that such stiffness matrix does not change with the applied macroscopic strain.
(ii) A NN is used to perform a classification exercise, to distinguish between elastic increments (in which no plastic dissipation occurs, $\Delta W_{pl} = 0$) from elastic–plastic increments (in which some energy is plastically dissipated, $\Delta W_{pl} > 0$).
(iii) Then, if an increment is classified as elastic, the change in the macroscopic stress state of the VE is obtained from the equations of linear elasticity, using the stiffness matrix calculated in step (i). Conversely, if an increment is classified as elastic–plastic, a second NN is used to perform a regression exercise to predict the change in the macroscopic stress state of the VE over that increment.

In the following, due to the isotropy of the VEs and the small strains imposed, we will assume (as observed from the simulations) that no substantial strain localisation and softening occur during deformation of the VEs, such that the macroscopic principal stress ${{{{{\varvec{\upsigma}}}}}}$ and principal strain ${{\varvec{\upvarepsilon}}}$ are, in first approximation, sufficient to describe the state of the solid at any given point in time, and that history effects can be quantified by appropriate auxiliary variables depending on the histories of ${{{{{\varvec{\upsigma}}}}}}$ and ${{\varvec{\upvarepsilon}}}$. This justifies the use of simple feed-forward NNs as opposed to more complex, computationally demanding, and hard to train recurrent NNs^13^.

We also note that the presence of the classification step can be in principle avoided, however in our preliminary studies we found that including the preliminary classification step increases the accuracy by approximately 20%.

We now proceed to describe the three different models proposed in this study.

##### Model I

The classification is performed by a neural network $NN_{clas}^{I}$ having as inputs ${{{{{\varvec{\upsigma}}}}}}$, ${{\varvec{\upvarepsilon}}}$ and $\Delta {{\varvec{\upvarepsilon}}}$ for each increment, and providing a single scalar output $NN_{clas}^{I} \left( {{\mathbf{\sigma ,\varepsilon ,}}\Delta {{\varvec{\upvarepsilon}}}} \right) \in \left[ {0,1} \right]$, such that an increment is considered elastic if $NN_{clas}^{I}$ has an output less than 0.5 and elastic–plastic otherwise. To train such NN we proceed broadly as described in “Training of the neural networks”. Our results database is pre-processed to include, for each increment, a column $y_{label}$ that is set to 0 if no plastic dissipation occurs over that increment, and to 1 otherwise

17 $$y_{label} = \left\{ {\begin{array}{*{20}l} 0 \hfill & {if\,\,\Delta W_{pl} = 0\,\,{\text{(elastic)}}} \hfill \\ 1 \hfill & {if\,\,\Delta W_{pl} > 0\,\,{\text{(elastic - plastic)}}} \hfill \\ \end{array} } \right.$$

(note that $\Delta W_{pl} < 0$ does not occur in our thermodynamically consistent training dataset).The desired number of training data points $\phi$ is selected from the database such that $\phi /2$ such points have $y_{label} = 0$ and $\phi /2$ have $y_{label} = 1,$ to avoid any bias. A number $\bar{\phi } = 0.9\phi$ of these data points is randomly selected for training and the remaining $0.1\phi$ is used for validation. The loss function used to train $NN_{clas}^{I}$ is chosen as the binary cross-entropy *BCE*, defined as

18 $$BCE = \frac{ - 1}{{\bar{\phi }}}\sum\limits_{j = 1}^{{\bar{\phi }}} {\left( {y_{label}^{j} \ln \left( {NN_{clas}^{I} \left( {\frac{{{{{{{\varvec{\upsigma}}}}}}^{j} }}{{\overline{E}}}{\mathbf{,\varepsilon }}^{j} {\mathbf{,}}\Delta {{\varvec{\upvarepsilon}}}^{j} } \right)} \right) + \left( {1 - y_{label}^{j} } \right)\ln \left( {1 - NN_{clas}^{I} \left( {\frac{{{{{{{\varvec{\upsigma}}}}}}^{j} }}{{\overline{E}}}{\mathbf{,\varepsilon }}^{j} {\mathbf{,}}\Delta {{\varvec{\upvarepsilon}}}^{j} } \right)} \right)} \right)}$$

with $j = 1,2..\bar{\phi }$ used to sum over each of the training data points.

The regression to predict the change in the macroscopic stress state is performed by a separate NN using the same inputs as $NN_{clas}^{I}$ and providing as output $\Delta {{{{{\varvec{\upsigma}}}}}}$, as

19 $$\Delta {{{{{\varvec{\upsigma}}}}}}/\overline{E} = NN_{reg}^{I} \left( {{{{{{\varvec{\upsigma}}}}}}/\overline{E}{\mathbf{,\varepsilon ,}}\Delta {{\varvec{\upvarepsilon}}}} \right).$$

This is the simplest possible choice to capture a path-dependent, rate-independent response, and it assumes that path-dependence is completely quantified by knowledge of the initial ${{{{{\varvec{\upsigma}}}}}}$ and ${{\varvec{\upvarepsilon}}}$.

##### Model II

In this model we include as an input for the classification and regression a macroscopic measure of the accumulated equivalent von Mises plastic strain; this is obtained by assuming that the total macroscopic strains can be decomposed as the sum of elastic and plastic parts

20 $${{\varvec{\upvarepsilon}}} = {{\varvec{\upvarepsilon}}}^{el} + {{\varvec{\upvarepsilon}}}^{pl} = {\mathbf{C}}_{0}^{ - 1} {{{{{\varvec{\upsigma}}}}}} + {{\varvec{\upvarepsilon}}}^{pl} ;\,\,\,\,\,{\mathbf{\Delta \varepsilon }} = {\mathbf{\Delta \varepsilon }}^{el} + {\mathbf{\Delta \varepsilon }}^{pl} = {\mathbf{C}}_{0}^{ - 1} {\mathbf{\Delta \sigma }} + {\mathbf{\Delta \varepsilon }}^{pl} .$$

The increments in a ‘macroscopic’ equivalent von Mises plastic strain are computed as

21 $$\Delta \bar{\varepsilon }_{0}^{pl} = \sqrt {\frac{2}{3}\Delta {{\varvec{\upvarepsilon}}}^{pl} \Delta {{\varvec{\upvarepsilon}}}^{pl} }$$

and its accumulated value $\bar{\varepsilon }_{0}^{pl}$ is computed as a function of time. In this model we prefer to use ${{\varvec{\upvarepsilon}}}^{el}$, ${{\varvec{\upvarepsilon}}}^{pl}$ in lieu of ${{{{{\varvec{\upsigma}}}}}}$, ${{\varvec{\upvarepsilon}}}$, as inputs for the NNs; the macroscopic response of the VE is expected to be elastic–plastic with a hardening response not necessarily isotropic, as prescribed at a local level. For this reason, we prefer to feed to the NNs the explicit knowledge of the plastic strains ${{\varvec{\upvarepsilon}}}^{pl} .$

The classification is performed by a neural network $NN_{clas}^{II}$ having as inputs ${{\varvec{\upvarepsilon}}}^{el}$, ${{\varvec{\upvarepsilon}}}^{pl}$, $\Delta {{\varvec{\upvarepsilon}}}$ and $\bar{\varepsilon }_{0}^{pl}$ for each increment, and providing a single scalar output $NN_{clas}^{II} \left( {{{\varvec{\upvarepsilon}}}^{el} ,{{\varvec{\upvarepsilon}}}^{pl} {\mathbf{,}}\bar{\varepsilon }_{0}^{pl} {\mathbf{,}}\Delta {{\varvec{\upvarepsilon}}}} \right) \in \left[ {0,1} \right]$, used to determine if an increment is elastic $\left( {NN_{clas}^{II} < 0.5} \right)$ or elastic–plastic $\left( {NN_{clas}^{II} \ge 0.5} \right)$. The loss function for the classification exercise is identical to that for *Model I*.

The regression is performed by a separate NN using the same inputs as $NN_{clas}^{II}$ and providing as an output $\Delta {{\varvec{\upvarepsilon}}}^{pl}$

22 $$\Delta {{\varvec{\upvarepsilon}}}^{pl} = NN_{reg}^{II} \left( {{{\varvec{\upvarepsilon}}}^{el} ,{{\varvec{\upvarepsilon}}}^{pl} {\mathbf{,}}\bar{\varepsilon }_{0}^{pl} {\mathbf{,}}\Delta {{\varvec{\upvarepsilon}}}} \right).$$

The corresponding increment in stress can readily be obtained from Eq. (20), ${\mathbf{\Delta \sigma }} = {\mathbf{C}}_{0}^{{}} \left( {{\mathbf{\Delta \varepsilon }} - {\mathbf{\Delta \varepsilon }}^{pl} } \right)$.

**Figure 3:**
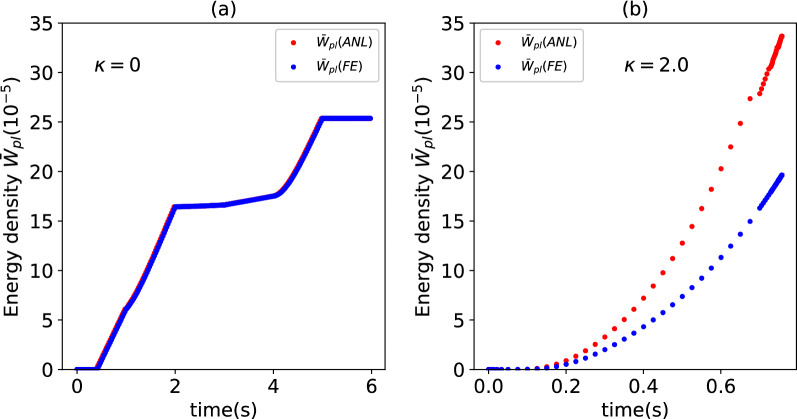
Comparison of the time histories of the density of plastically dissipated energy during selected random multiaxial loading cases, as calculated analytically from Eq. (23) (label *ANL*) or obtained from the FE simulations (label *FE*). The comparison is made for a homogeneous volume element ((**a**), $\kappa = 0$) and for a heterogeneous material with spatially varying Young’s modulus $\left( {\kappa = 2} \right)$ and uniform plastic properties (**b**).

We note here that decomposing the macroscopic strains into an elastic and plastic component is not, strictly, physically consistent. For example, one cannot use knowledge of macroscopic stress and macroscopic plastic strain to compute the density of plastically dissipated energy in the volume element, i.e.

23 $${{{{{\varvec{\upsigma}}}}}}\Delta {{\varvec{\upvarepsilon}}}^{pl} \ge \Delta W_{pl}^{FE}$$

where $\Delta W_{pl}^{FE}$ is the density in plastically dissipated energy computed by the FE simulations; this is because the left-hand side of the inequality, ${{{{{\varvec{\upsigma}}}}}}\Delta {{\varvec{\upvarepsilon}}}^{pl}$, in the case of heterogeneous solids, includes the plastic-free energy, which is the elastic strain energy stored in the elastic regions of a plastically deforming heterogeneous volume element^24^. The inequality (23) violates the Hill-Mendel condition^3^ unless the equal sign applies. This is illustrated in Fig. 3, where we compare the plastically dissipated energy calculated from the knowledge of the macroscopic plastic strains (as $W_{pl} = \int_{0}^{t} {{{{{{\varvec{\upsigma}}}}}}\,\left( {d{{\varvec{\upvarepsilon}}}^{pl} /dt} \right)dt}$) to that determined by the FE simulations. The plastically dissipated energy is normalised in Fig. 3 as $\overline{W}_{pl} = W_{pl} /\left( {\overline{E}V_{0} } \right)$, and evaluated in the case of a homogeneous volume element (for which ${{{{{\varvec{\upsigma}}}}}}\Delta {{\varvec{\upvarepsilon}}}^{pl} = \Delta W_{pl}^{FE}$, Fig. 3a) and for a composite of heterogeneity $\kappa = 2$, for which ${{{{{\varvec{\upsigma}}}}}\Delta {\varvec{\upvarepsilon}}}^{pl} \ge \Delta W_{pl}^{FE}$ (Fig. 3b).

##### Model III

To avoid the physical inconsistency of *Model II*, we derive an alternative model which does not make use of the accumulated macroscopic equivalent von Mises plastic strain $\bar{\varepsilon }_{0}^{pl}$ or of the macroscopic plastic strain components $\Delta {{\varvec{\upvarepsilon}}}^{pl}$. In *Model III* we replace $\bar{\varepsilon }_{0}^{pl}$ by $W_{pl}^{{}}$ as an input of the NNs and we perform an additional regression to predict $\Delta W_{pl}^{{}}$ at every increment.

A classification to distinguish elastic from elastic–plastic increments if performed by a neural network $NN_{clas}^{III} \left( {{{\varvec{\upvarepsilon}}},{{{{{\varvec{\upsigma}}}}}}/\overline{E}{\mathbf{,}}\overline{W}_{pl} {\mathbf{,}}\Delta {{\varvec{\upvarepsilon}}}} \right) \in \left[ {0,1} \right]$, where $\overline{W}_{pl} = W_{pl} /\left( {\overline{E}V_{0} } \right)$; the two regressions described above use NNs with the same inputs as $NN_{clas}^{III}$, namely

24 $$\Delta {{{{{\varvec{\upsigma}}}}}}/\overline{E} = NN_{{reg_{\sigma } }}^{III} \left( {{{\varvec{\upvarepsilon}}},{{{{{\varvec{\upsigma}}}}}}/\overline{E}{\mathbf{,}}\overline{W}_{pl} {\mathbf{,}}\Delta {{\varvec{\upvarepsilon}}}} \right),$$

25 $$\Delta \overline{W}_{pl} = NN_{{reg_{W} }}^{III} \left( {{{\varvec{\upvarepsilon}}},{{{{{\varvec{\upsigma}}}}}}/\overline{E}{\mathbf{,}}\overline{W}_{pl} {\mathbf{,}}\Delta {{\varvec{\upvarepsilon}}}} \right).$$

As for the previous models, the chosen loss functions are *BCE* for the classification and *MAE* for the two regressions.

### Using the NNs to predict stress versus strain histories

Once successfully trained, the NNs are used as surrogate models to predict the homogenised constitutive response of the VEs subject to previously unseen, pseudo-random, multiaxial macroscopic strain histories. To assess the accuracy of each surrogate model we generate 20 additional unseen loading cases, in the form of pseudo-random walks in true principal strain space, as described in “Generation of the training datasets”. These loading cases are applied to the same realisation of the VE used to produce the training data, and the corresponding simulations are conducted in Abaqus Standard as in “Finite element simulations”. The macroscopic stress versus strain histories are extracted from the simulations and used as a benchmark for the surrogate models.

The predictions by the surrogate model are obtained as follows. First, macroscopic strains and stresses are initialised and set to zero. Then, we consider the first increment of the simulations, corresponding to time and strain increments $\Delta t_{1}^{FE}$, $\Delta {{\varvec{\upvarepsilon}}}_{1}^{FE}$; the NNs described in the previous section are used to predict the stress and strain vectors at the end of the first increment, using $\Delta t_{1}^{FE}$, $\Delta {{\varvec{\upvarepsilon}}}_{1}^{FE}$ as inputs. Such updated stress and strain vectors are used as inputs of the NNs for the following increment; such NNs always use time and strain increments identical to those in the FE solution, $\Delta t = \Delta t_{{}}^{FE} ,$ $\Delta {{\varvec{\upvarepsilon}}} = \Delta {{\varvec{\upvarepsilon}}}_{{}}^{FE} .$ Repeating this procedure iteratively, we construct predictions of the stress versus strain curves using the surrogate models.

Generating a prediction of the stress versus strain history for an average random loading case takes approximately less than one second of CPU time. We note that obtaining the corresponding predictions via FE simulations requires approximately 350 s, therefore the surrogate models offer a computational saving of at least two orders of magnitudes. In this study, the VE was meshed by only 10^3^ elements, but this number was kept low in the interest of speed. For very detailed VE of materials with complex architecture, such a number can easily exceed 10^8^, corresponding to a great increase in the CPU time required to perform the FE simulations; however, the time required by the surrogate models to produce the same predictions would remain below 1 s. Also, in this study we use NNs with 100 neurons per layer; the number of neurons can be reduced substantially without a corresponding reduction in accuracy, as it was found in a preliminary study not reported here for brevity.

### Quantification of the accuracy of the surrogate models

To assess the accuracy of the regressions performed by the NNs $$NN_{reg}^{I} ,$$ $$NN_{reg}^{II} ,$$ $$NN_{{reg_{\sigma } }}^{III} ,$$$$NN_{{reg_{W} }}^{III} ,$$ in addition to the final value of the loss function *MAE*, we introduce an additional metric, to which we refer as *path-wise stress error*, $$E_{{{{{{\varvec{\upsigma}}}}}}}$$; this metric aims at capturing the effectiveness of a surrogate model in predicting macroscopic stress versus strain histories of a VE. This is computed as follows. A pseudo-random loading case is generated as described in “Generation of the training datasets” and the corresponding FE simulation (comprising $$N_{inc}$$ increments) is conducted. The macroscopic stress predicted by the detailed FE simulation, referred to as $${{{{{\varvec{\upsigma}}}}}}_{FE}$$ here, is compared to the corresponding prediction by a NN (at the same strain $${{\varvec{\upvarepsilon}}}$$), indicated as $${{{{{\varvec{\upsigma}}}}}}_{NN}$$, at the end of every increment, and $$E_{{{{{{\varvec{\upsigma}}}}}}}$$ is defined as

26 $$E_{\varvec{\sigma }} = \frac{1}{{N_{{inc}} }}\sum\limits_{{j = 2}}^{{N_{{inc}} }} {\left( {\frac{{\left\| {\varvec{\sigma }_{{NN_{j} }} - \varvec{\sigma }_{{FE_{j} }} } \right\|_{2} }}{{\left\| {\varvec{\sigma }_{{FE_{j} }} } \right\|_{2} }}} \right)}$$

where *j* is used to sum over different increments and $\left\| * \right\|_{2}$ denotes the norm 2 of *. To compare the accuracy of different models, $E_{{{{{{\varvec{\upsigma}}}}}}}$ is evaluated over 20 different pseudo-random loading cases and the corresponding average $\overline{E}_{{{{{{\varvec{\upsigma}}}}}}}$ is computed.

When assessing the effectiveness of the classification exercises performed in this study by $NN_{clas}^{I} ,$ $NN_{clas}^{II} ,$ $NN_{clas}^{III} ,$ we define a *path-wise accuracy* ${\text{ A}}$ as

27 $${\rm A} = \frac{1}{{N_{inc} }}C_{correct}$$

where $C_{correct}$ is the total number of correct classification instances over a certain random loading case. Similarly to the case of the regressions, this is computed for a population of 20 different loading cases and the average $\overline{\text{ A}}$ is evaluated.

## Results and discussion

We proceed to present the predictions of the surrogate models for each constitutive description of the n-phase composites analysed. We note here that the predictions shown below, obtained with accurately trained NNs, were found to be thermodynamically consistent, with no exception, in the sense that in all cases, and in all increments of the simulations conducted in this study, the energy was conserved and the dissipated energy was non-negative. This can be explained by the fact that our training data is thermodynamically consistent and that the NN have an excellent agreement with this data at the end of the training. During the training/optimisation of the NNs, it can occur that thermodynamically inconsistent predictions are made, especially at the early stages of the optimisation. In this study, however, this problem was not detected after the training, such that it was not necessary to include explicit constraints in the optimisation problem.

We also checked, by appropriate permutations of the indices of inputs and outputs along the three Cartesian directions, that the predictions of the surrogate models were approximately isotropic as expected.

### Hyperelastic n-phase composite

**Figure 4:**
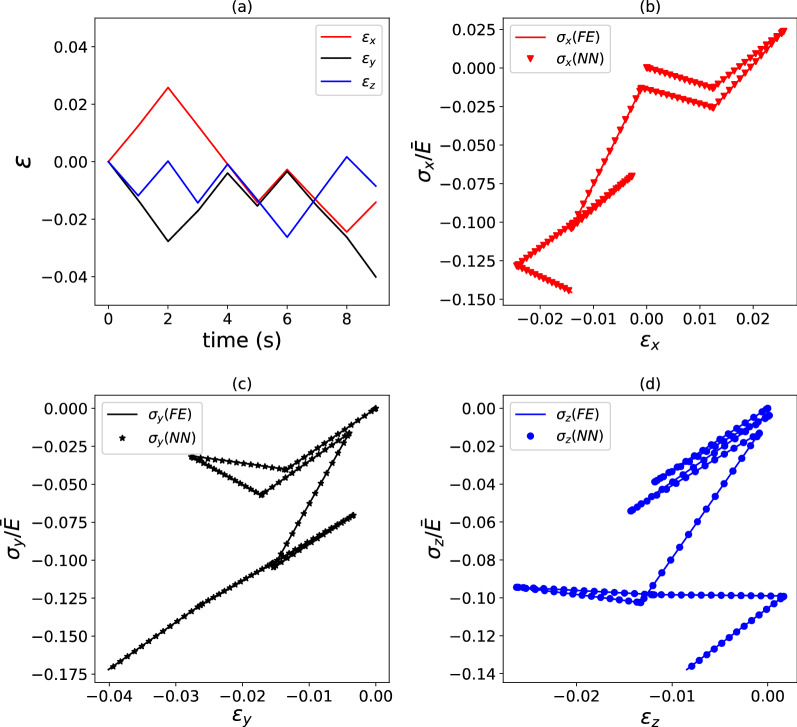
Example of strain versus time (**a**) and stress versus strain (**b**–**d**) histories for a hyperelastic composite with $\kappa = 2.0.$ FE predictions are compared to predictions by a surrogate model. $E_{{{{{{\varvec{\upsigma}}}}}}} = 1.7\%$ for the load case shown, which used $\phi = 4000$ training data points.

We recall that these composites comprise an array of $10 \times 10 \times 10$ cubic cells (each meshed by a single FE of type C3D8 in Abaqus^18^) with dissimilar Young’s moduli and uniform Poisson’s ratio as detailed in “Computational framework”. Figure 4 presents, for a selected pseudo-random loading case, a comparison of the predictions of the FE simulations and of the surrogate model. These are found in excellent agreement and correspond to a path-wise stress error $E_{{{{{{\varvec{\upsigma}}}}}}} = 1.7\%$. $\phi = 4000$ datapoints were used to train the surrogate model.

**Figure 5:**
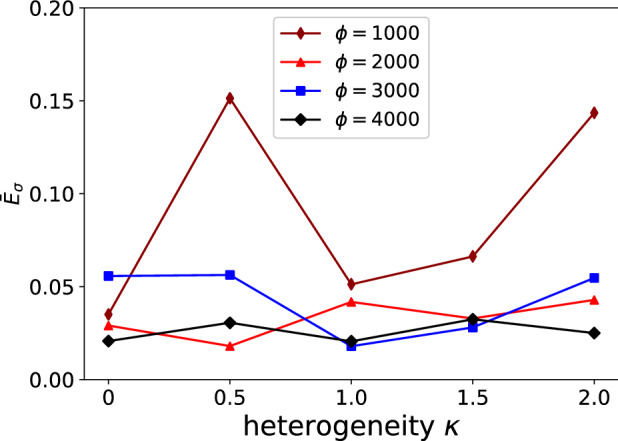
Average path-wise stress error $\overline{E}_{{{{{{\varvec{\upsigma}}}}}}}$ (calculated over 20 different pseudo-random loading paths) as a function of the heterogeneity $\kappa$ and the number of training datapoints $\phi .$

Considering the small strains imposed on the composite, we expect the NN (12) to predict comfortably the correspondence between stress and strain, which is very close to a linear elastic relation. Figure 5 shows that this is indeed the case. Only 2000 training data points are required to ensure $E_{{{{{{\varvec{\upsigma}}}}}}} < 5\%$; the surrogate model shows accuracy approximately independent of the degree of heterogeneity $\kappa$.

We note that choosing a NN with a structure like in (12), rather than looking for an elastic potential as pursued by other authors (e.g.^25^), has the advantage of being suitable in cases where such elastic potential does not exist.

### Viscoelastic n-phase composite

**Figure 6:**
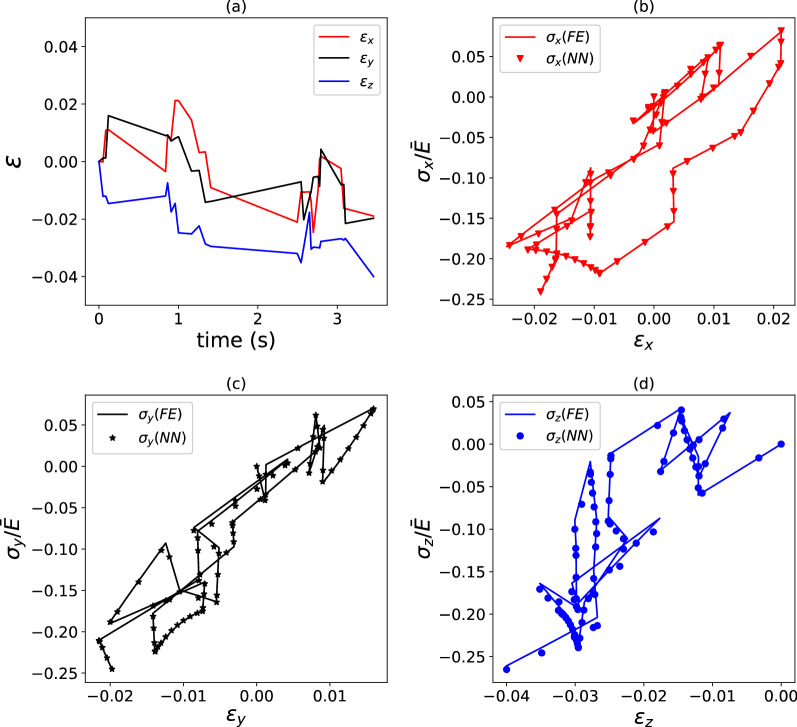
Example of strain versus time (**a**) and stress versus strain (**b**–**d**) histories for a viscoelastic composite with $\kappa = 2.0$ and stiffness relaxation dictated by a single-term Prony series. FE predictions are compared to predictions by a surrogate model. $E_{{{{{{\varvec{\upsigma}}}}}}} = 6.2\%$ for the load case shown, which used $\phi = 5000$ training data points.

**Figure 7:**
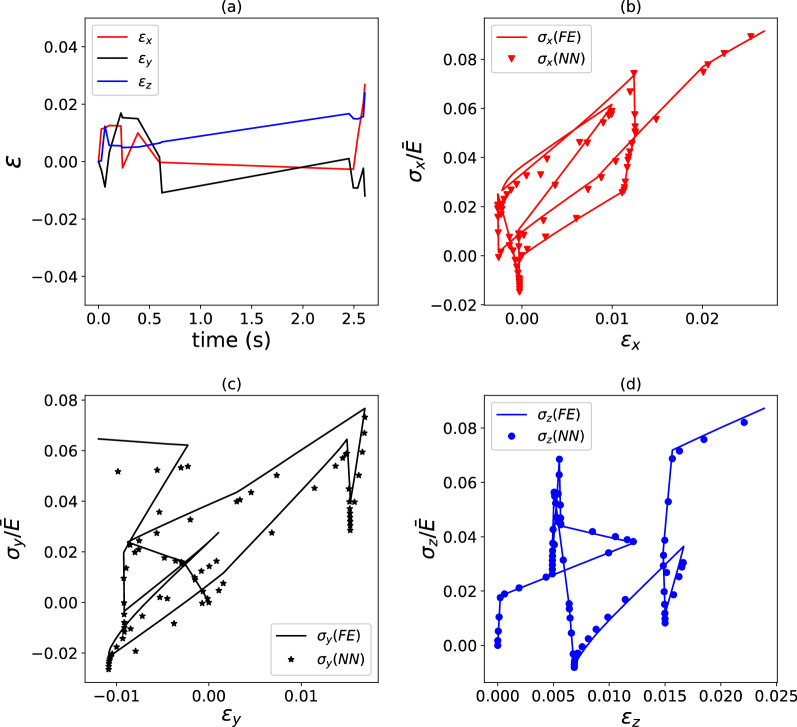
Example of strain versus time (**a**) and stress versus strain (**b**–**d**) histories for a viscoelastic composite with $\kappa = 2.0$ and stiffness relaxation dictated by a three-term Prony series. FE predictions are compared to predictions by a surrogate model. $E_{{{{{{\varvec{\upsigma}}}}}}} = 19.4\%$ for the load case shown, which used $\phi = 5000$ training data points.

We proceed to assess the accuracy of the surrogate model of a viscoelastic composite. Figure 6 compares the FE predictions to those by the surrogate model for a composite with relaxation characteristic given by a single-term Prony series, while Fig. 7 presents the same information for the case of a relaxation function comprising a three-term Prony series. We note that for both cases $\eta_{\min }$ = − 3, $\eta_{\max }$ = 0, such that the strain rates in each increment are logarithmically spread over three orders of magnitude and comprised between $10^{ - 3}$ and $10^{0} \,{\text{s}}^{{ - {1}}} .$ We found in a preliminary study that logarithmic spacing of the training data points was more effective than linear spacing.

**Figure 8:**
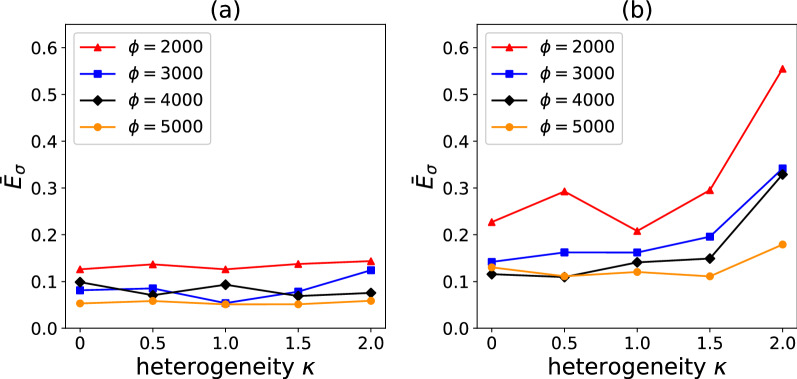
Average path-wise stress error $\overline{E}_{{{{{{\varvec{\upsigma}}}}}}}$ (calculated over 20 different pseudo-random loading paths) as a function of the heterogeneity $\kappa$ and the number of training data points $\phi$. Data are shown for a viscoelastic model using a single-term Prony series (**a**) or a three-term Prony series (**b**).

The data in Figs. 6 and 7 correspond to $\kappa = 2$ and $\phi = 5000$. For this choice, the surrogate model performs an excellent regression for a composite with a single-term Prony series, achieving a path-wise stress error $E_{{{{{{\varvec{\upsigma}}}}}}} = 6.2\%$. In the case of a relaxation function comprising a three-term Prony series, the accuracy of the surrogate model is lower but still acceptable $\left( {E_{{{{{{\varvec{\upsigma}}}}}}} = 19.4\% } \right)$, especially in consideration of the very complex loading case imposed on the composite. The accuracy of the regressions could be improved by increasing the number of training datapoints, but this is not investigated here. Figure 8 provides average values of $\overline{E}_{{{{{{\varvec{\upsigma}}}}}}} ,$ calculated over 20 different pseudo-random loading cases, for the two composites examined. Clearly, the accuracy of the model is relatively insensitive to the degree of heterogeneity for the case of a single-term Prony series (Fig. 8a); for a three-term Prony series (Fig. 8b), the surrogate models give lower $\overline{E}_{{{{{{\varvec{\upsigma}}}}}}}$ at low heterogeneity $\kappa$, but the error becomes less and less sensitive to $\kappa$ with an increasing number of training data points $\phi .$ The trends in Fig. 8a suggest that the error can be lowered by increasing the number of training data points.

We conclude that our proposed regression (15) is effective in capturing the response of volume elements with a general linear viscoelastic response, loaded over wide ranges of strains and strain rates. The proposed method directly provides macroscopic stresses and strains without the need for defining a “macroscopic relaxation function” as pursued in other studies^26^.

### Elastic–plastic n-phase composite

**Figure 9:**
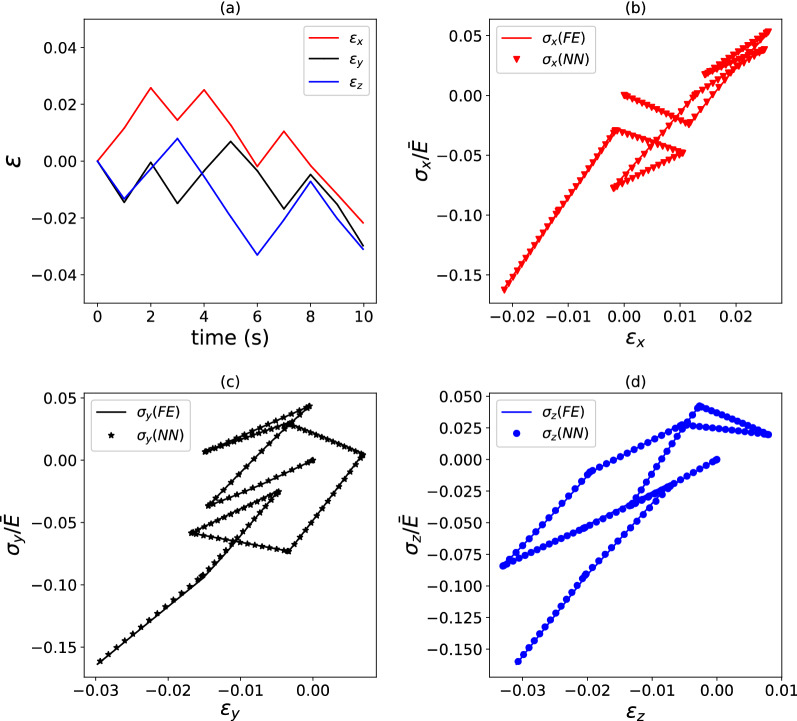
Example of strain versus time (**a**) and stress versus strain (**b**–**d**) histories for an elastic–plastic composite with $\kappa = 2.0$. FE predictions are compared to predictions by a surrogate model. $E_{{{{{{\varvec{\upsigma}}}}}}} = 2.7\%$ for the load case shown, which used $\phi = 5000$ training data points.

**Figure 10:**
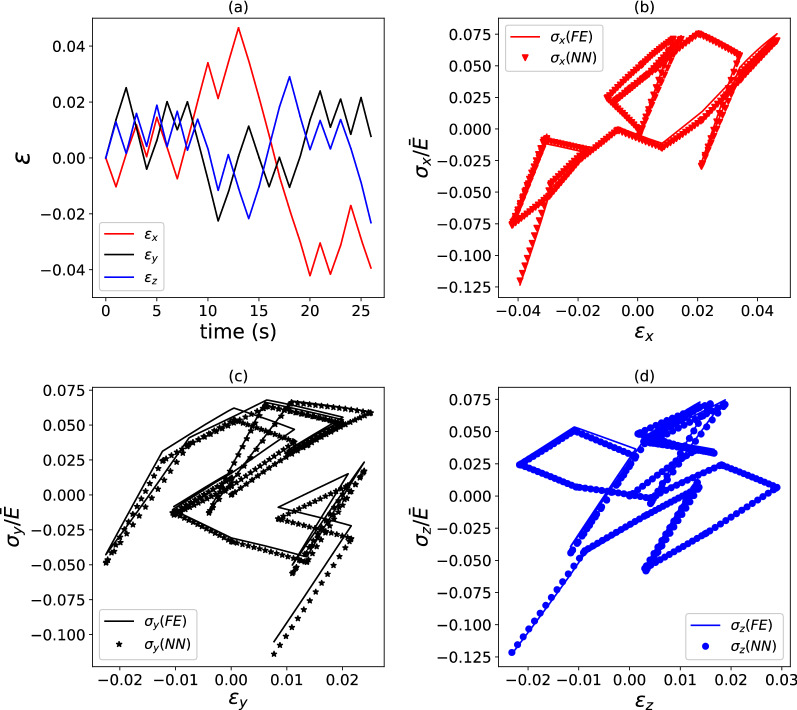
Example of strain versus time (**a**) and stress versus strain (**b**–**d**) histories for an elastic–plastic composite with $\kappa = 2.0$. FE predictions are compared to predictions by a surrogate model. $E_{{{{{{\varvec{\upsigma}}}}}}} = 17.8\%$ for the load case shown, which used $\phi = 5000$ training data points.

We now examine the accuracy of the surrogate models proposed for the elastic–plastic composites; in this section, we only focus on composites with heterogeneous Young’s modulus and uniform yield stress, while the case of uniform modulus and spatially varying yield stress is presented in Appendix B (supplementary material) for the sake of brevity. The predictions of Model II are compared to the results of FE simulations in Figs. 9 and 10, in terms of stress versus strain histories, for the highest possible heterogeneity, $\kappa = 2.$ We choose to plot the predictions of Model II because this model outperforms the other two, as it will be shown below. Figures 9 and 10 correspond to selected pseudo-random loading paths, with Fig. 9 referring to a relatively simple path and Fig. 10 present the case of a much more complicated path. In both cases, Model II achieves excellent accuracy, however, as expected, this accuracy is higher for the simpler loading path $\left( {E_{\sigma } = 2.7\% } \right)$ than for the more complicated one $\left( {E_{\sigma } = 17.8\% } \right)$.

**Figure 11:**
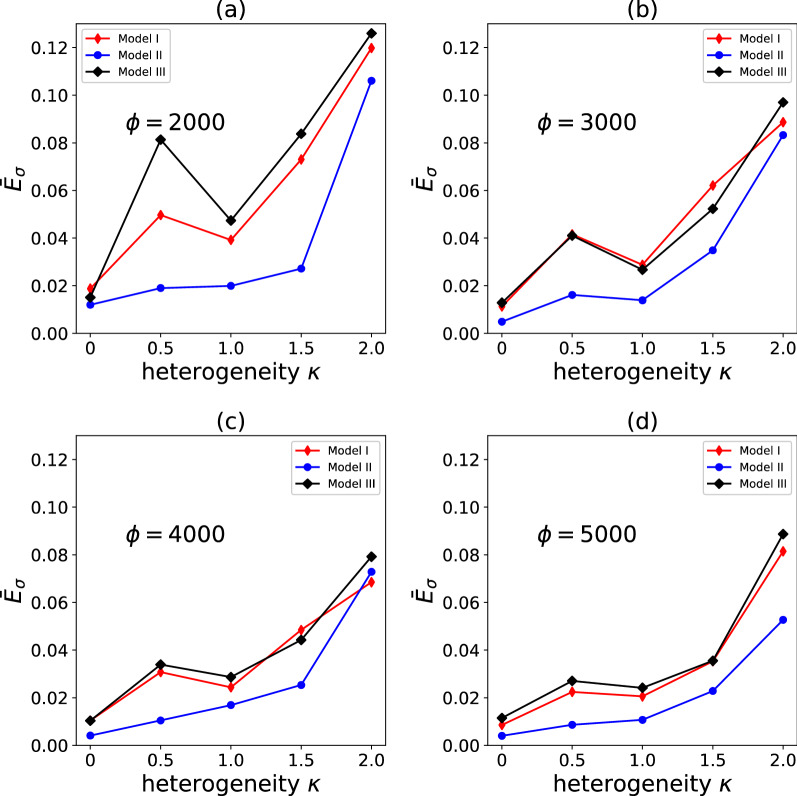
Average path-wise stress error $\overline{E}_{{{{{{\varvec{\upsigma}}}}}}}$ (calculated over 20 different pseudo-random loading paths) as a function of the heterogeneity $\kappa$ and the number of training data points $\phi$. Data are shown for models I, II and III.

Figure 11 compares the accuracy of Models I, II and III (in terms of the average path-wise stress error $\overline{E}_{\sigma }$) and the sensitivity of such accuracy to the degree of heterogeneity $\kappa$ and to the size of the training dataset $\phi .$ For $\phi = 5000$, Model II outperforms Model I, which in turn is more accurate than Model III; this trend is also observed at lower values of $\phi$. The accuracy of Model II is scarcely sensitive to the value of $\phi$, suggesting that $\phi = 2000$ is a sufficiently large dataset for the problem at hand. The general trend observed is that the accuracy of all models decreases as $\kappa$ increases.

**Figure 12:**
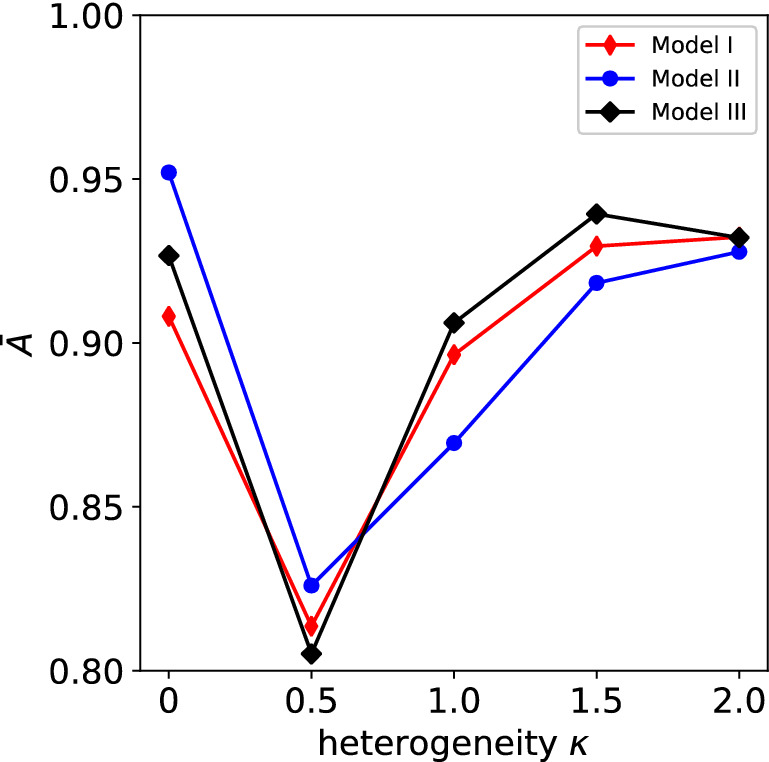
Average path-wise accuracy $\overline{A}$ (calculated over 20 different pseudo-random loading paths) as a function of the heterogeneity $\kappa$. The performance of the classifications in models I, II and III are compared.

**Figure 13:**
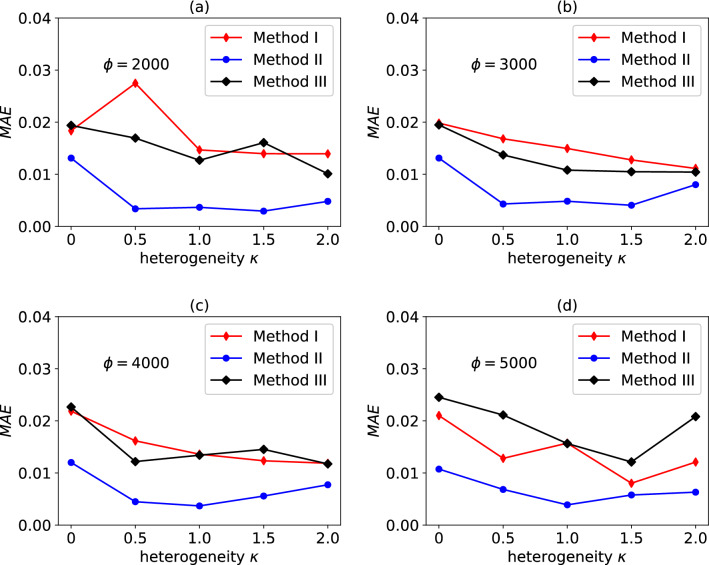
*MAE* for the validation dataset at convergence, for the NNs (19), (22) and (24), as a function of the heterogeneity $\kappa$ and the number of training data points $\phi$. Data are shown for models I, II and III.

The effectiveness of the classifications and regressions performed by Models I, II and III, are assessed independently in Figs. 12 and 13, respectively. Figure 12 presents the accuracy of the classification NNs used for the three models. Such accuracies are comparable for the three models, with Model III outperforming slightly the other two. Perhaps counter-intuitively, for $\kappa > 0$, the accuracy of the classification exercise is higher for a higher degree of heterogeneity. This is due to the fact that for very heterogeneous materials, the vast majority of the input space corresponds to plastic increments. We recall that the training dataset for these classifications contains equal numbers of data points in the plastic and elastic regimes, such that at high heterogeneity, data points that correspond to elastic increments tend to cluster in input space, and this makes the classification process easier for the NNs. Similarly, in the case $\kappa = 0$ a vast majority of increments are elastic, resulting in an easier classification.

In Fig. 13 we compare the values of *MAE* calculated for the validation dataset, for different values of $\kappa$ and $\phi$. We note that this quantity is independent of the length and complexity of the loading path and of the classification process, and only quantifies the effectiveness of the models in predicting the evolution of state variables over a single (elastic–plastic) increment. Model II always outperforms the other two irrespective of $\kappa$ and $\phi$; in absolute terms, the performance of Model II is nearly independent of $\phi$, again suggesting that $\phi = 2000$ is a sufficiently large dataset. At low $\phi$, Model III outperforms Model I, but the opposite is observed at high values of $\phi$.

In summary, Model III is the best of the three in the classification exercise, while Model II outperforms the other two models in the regression exercise (with models I and III approximately equivalent in regression performance). Overall, Model II is the best performing model in terms of $\overline{E}_{\sigma }$ and requires a smaller size of the training dataset to achieve a given accuracy. As larger and larger training datasets are employed, the performances of the three models, however (in terms of $\overline{E}_{\sigma }$) tend to become very similar. For the n-phase composites considered in this study, $\phi \ge 3000$ guarantees $\overline{E}_{\sigma } < 10\%$ for all models and irrespective of $\kappa$, which results in sufficiently accurate predictions in all cases of practical interest.

It is expected that composites with heterogeneous Young’s modulus and uniform yield stress (whose performance is discussed here and shown in Figs. 9, 10, 11, 12, and 13) display a macroscopic plastic response which is substantially pressure-sensitive; in contrast, composites with uniform Young’s modulus and spatially varying yield stress are expected to have an approximately pressure-insensitive macroscopic behaviour. Consequently, capturing the material response is expected to be easier in the second case. We found that indeed this is the case. Results for composites with uniform Young’s modulus and varying yield stress are presented in detail in Appendix B (supplementary material) for the benefit of the interested reader.

Some unexpected non-monotonic trends are observed in Figs. 11, 12 and 13; the reasons for these trends were not investigated further in this study, but these could be due to different distributions of training datapoints across the input space, for different values $\kappa$. Similarly, the relative performance of models II and III was also unexpected, in consideration of the slight physical inconsistency of Model II. While for the n-phase composites investigated here Model II outperforms (however slightly) Model III, this might not be the case for other types of heterogeneous materials containing plastically deforming phases. A detailed analysis of the relative performance of these two models and possible improvements to both is left as a topic for future studies.

## Concluding remarks

We proposed and assessed a computational framework to derive data-driven surrogate models for the response of non-linear n-phase composites to arbitrary mechanical loading. We considered composites with different non-linear local material responses, including non-linear elastic, time-dependent linear viscoelastic, and history-dependent elastic–plastic; in each case, we determine the macroscopic mechanical response via FE simulations and computational homogenisation and use data from the simulations to train appropriate neural networks to reproduce the homogenised response accurately. For each local constitutive description, we propose a computational procedure to produce training data and appropriate inputs and outputs for the surrogate models. We examine systematically the accuracy of the proposed surrogate models and we explore the sensitivity of their accuracy to the degree of heterogeneity of the composites and to the size of the training dataset. The main conclusions of the study are as follows:

- The strategy proposed to produce the training dataset, consisting of imposing pseudo-random histories of macroscopic deformation to the volume elements, results in training data representative of realistic scenarios for all three constitutive descriptions analysed in this study. The datasets produced appropriately sample the input space of the surrogate models, facilitating the application of machine learning techniques.
- For the type of loading examined in this study, restricted to small strains and negligible localisation of the stress, strain and strain rate fields, the proposed surrogate models proved very effective in capturing the non-linear multi-axial material response over wide ranges of applied strain rates and strain triaxiality, including non-monotonic and non-proportional loading.
- For elastic–plastic n-phase composites, absent any substantial strain localisation, the history-dependence of the material can be quantified adequately by knowledge of the current macroscopic stresses and strains; on the other hand, including appropriate history-dependent internal variables improves the accuracy of the surrogate models and decreases the size of the training dataset needed to achieve a prescribed accuracy.

The findings of this study may serve as a guide to researchers developing multi-scale computational models or data-driven models informed directly from measured data. Several improvements could be introduced to the proposed computational framework, such as: refining the features of the surrogate models, by including additional internal variables to aid capturing of the time- and history-dependence; relaxing the hypothesis of small strains and approximately isotropic response; extending the analysis to solids displaying a cohesive response and the consequent macroscopic softening and strain localisation. These improvements will be the subject of our future studies.

## Supplementary Information

Supplementary Information.
