# Supplementary material for: A computational framework to establish data-driven constitutive models for time- or path-dependent heterogeneous solids
Source: Sci Rep. 2021 Aug 5;11:15916. doi: 10.1038/s41598-021-94957-0 (PMC8342494; doi:10.1038/s41598-021-94957-0)
Supplement: Supplementary file 1 — Supplementary Information. [file 41598_2021_94957_MOESM1_ESM.docx]

**APPENDIX A: detailed analysis of the training dataset**

In this Appendix we present the details of the distributions of the training data used in this study across the input space, as specified in the figure captions. In all cases, the data shows an adequate spread over such space, and this contributes to the effectiveness of the machine learning exercise presented in this paper. In all cases, data is shown for the choice $\kappa=2.0$, . When visualising data in the three-dimensional Cartesian space, figures include a sphere of radius R as indicated in the figures, to quantify the magnitude of the data shown.


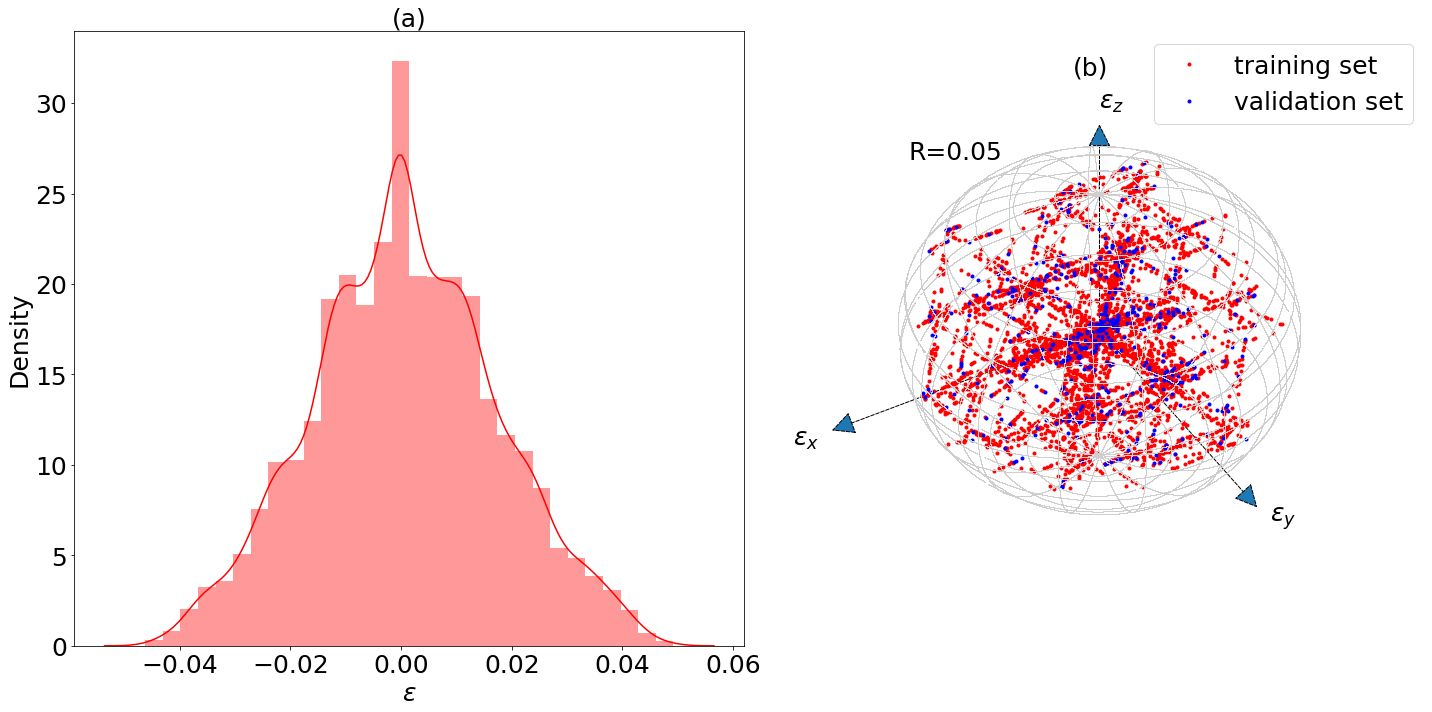


Figure A1. Probability density of the components of $\mathbf{\varepsilon}$(a) and visualisation of the same components in the three Cartesian directions (b), for the case of hyperelastic composites.


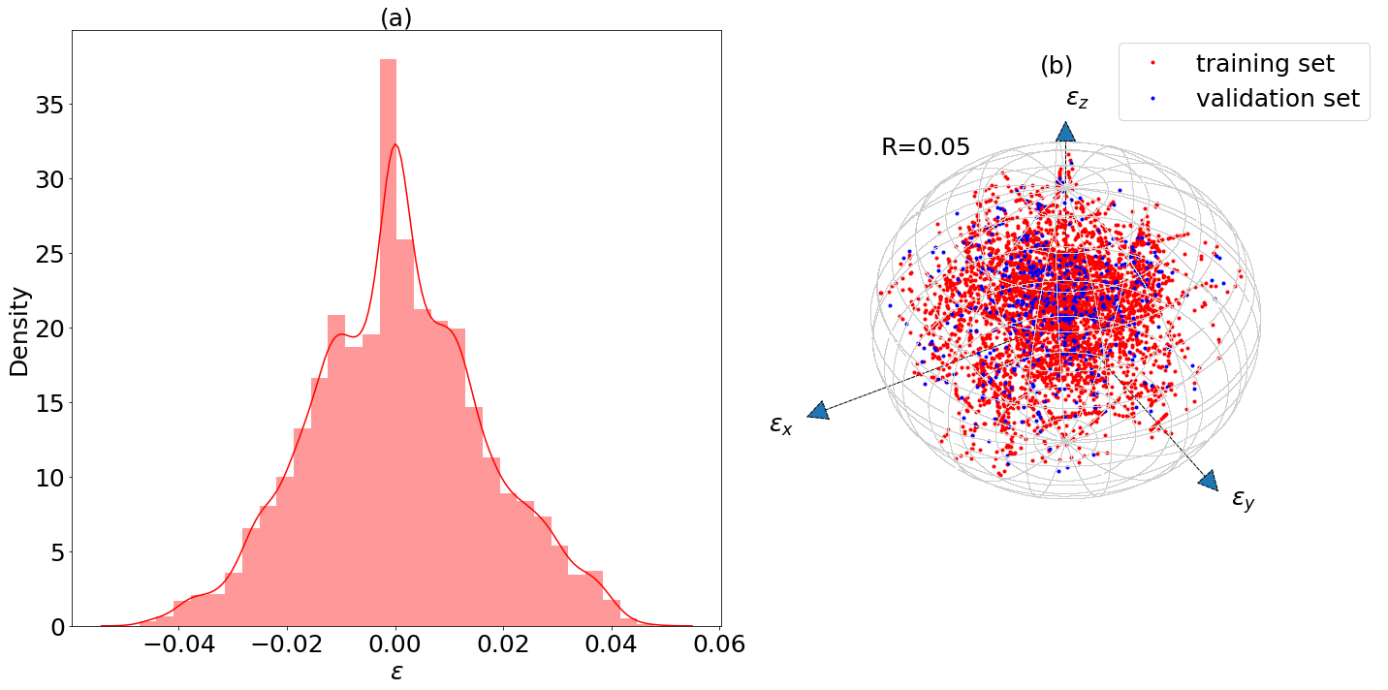


Figure A2. Probability density of the components of $\mathbf{\varepsilon}$(a) and visualisation of the same components in the three Cartesian directions (b), for the case of viscoelastic composites with relaxation expressed by a single-term Prony series.


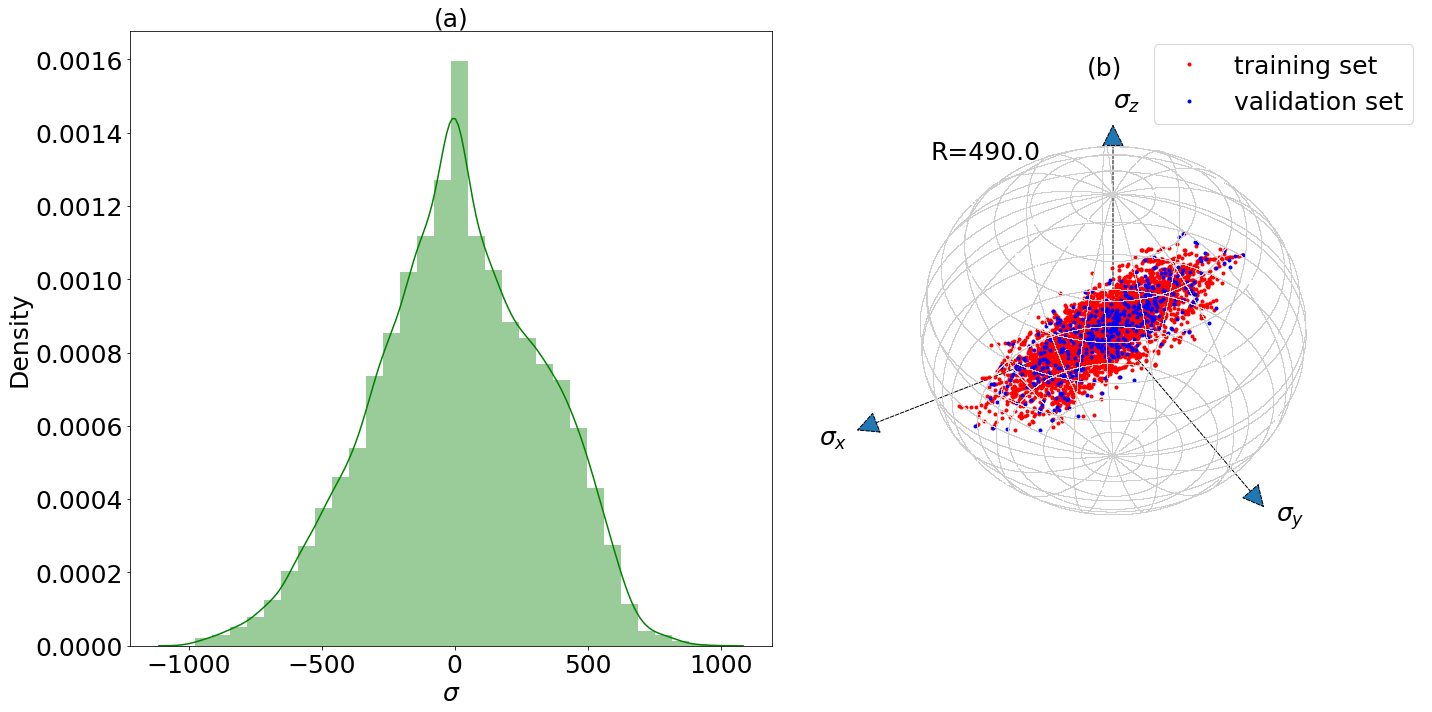


Figure A3. Probability density of the components of $\mathbf{\sigma}$(a, expressed in MPa) and visualisation of the same components in the three Cartesian directions (b), for the case of viscoelastic composites with relaxation expressed by a single-term Prony series.


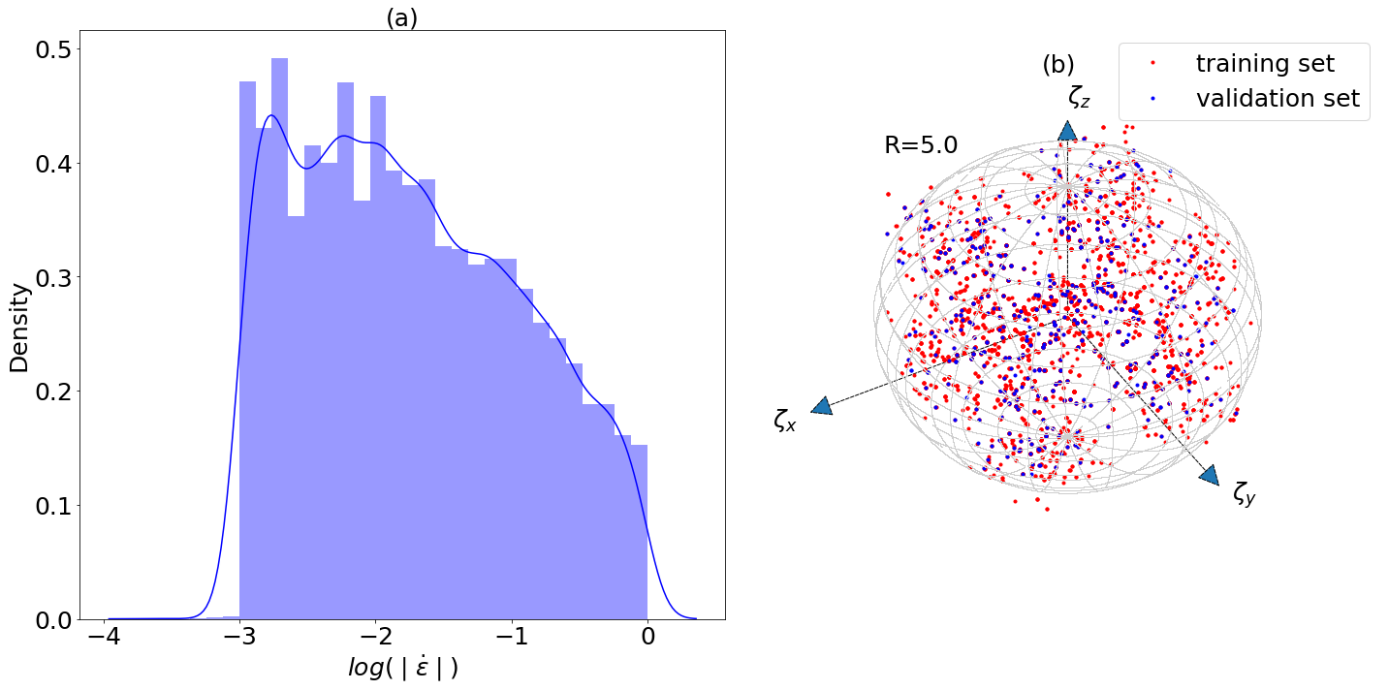


Figure A4. Probability density of the logarithm of the absolute value of the components of (a, with the components having units of s^-1^) and visualisation of the same components in the three Cartesian directions (b), for the case of viscoelastic composites with relaxation expressed by a single-term Prony series. In part (b) we plot , to preserve information about the sign of the strain rate components.


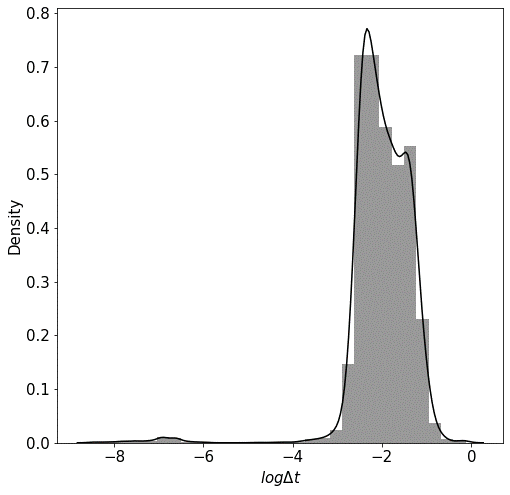


Figure A5. Probability density of $\log\left(\Delta t\right)$ (with $\Delta t$ expressed in seconds) for the case of viscoelastic composites with relaxation expressed by a single-term Prony series.


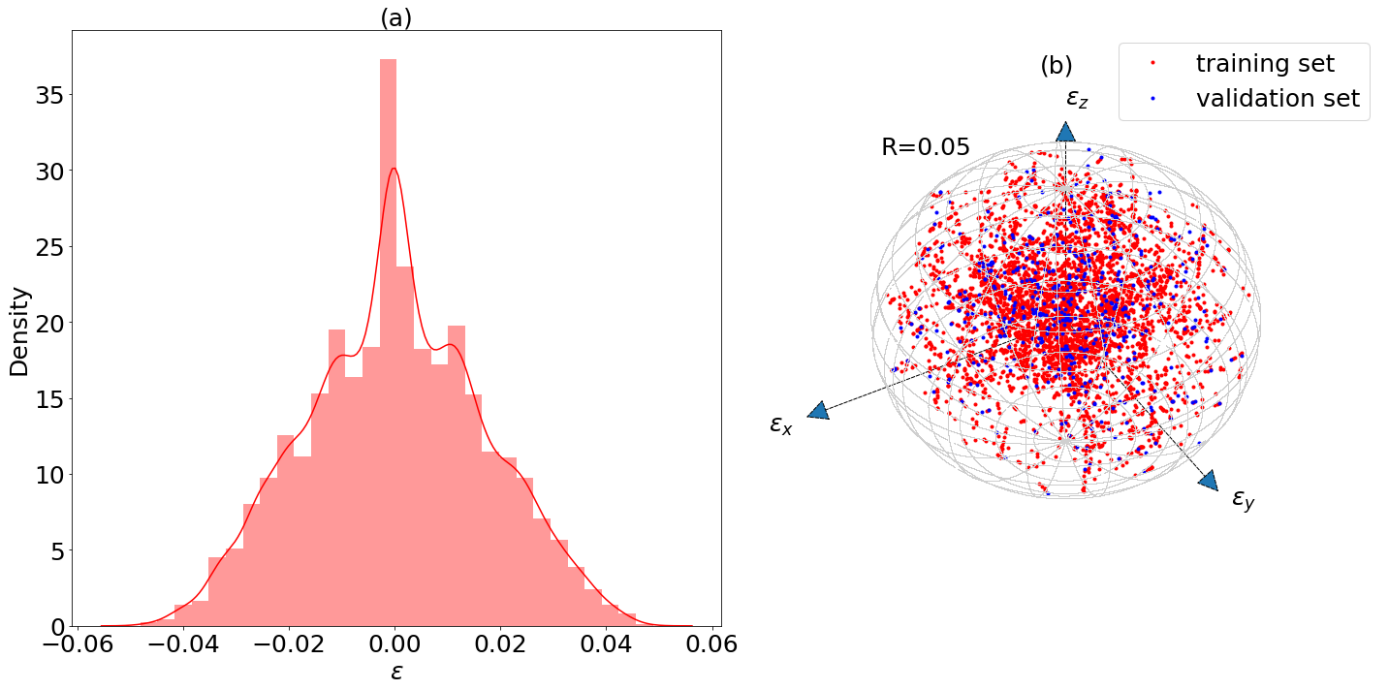


Figure A6. Probability density of the components of $\mathbf{\varepsilon}$(a) and visualisation of the same components in the three Cartesian directions (b), for the case of viscoelastic composites with relaxation expressed by a three-term Prony series.


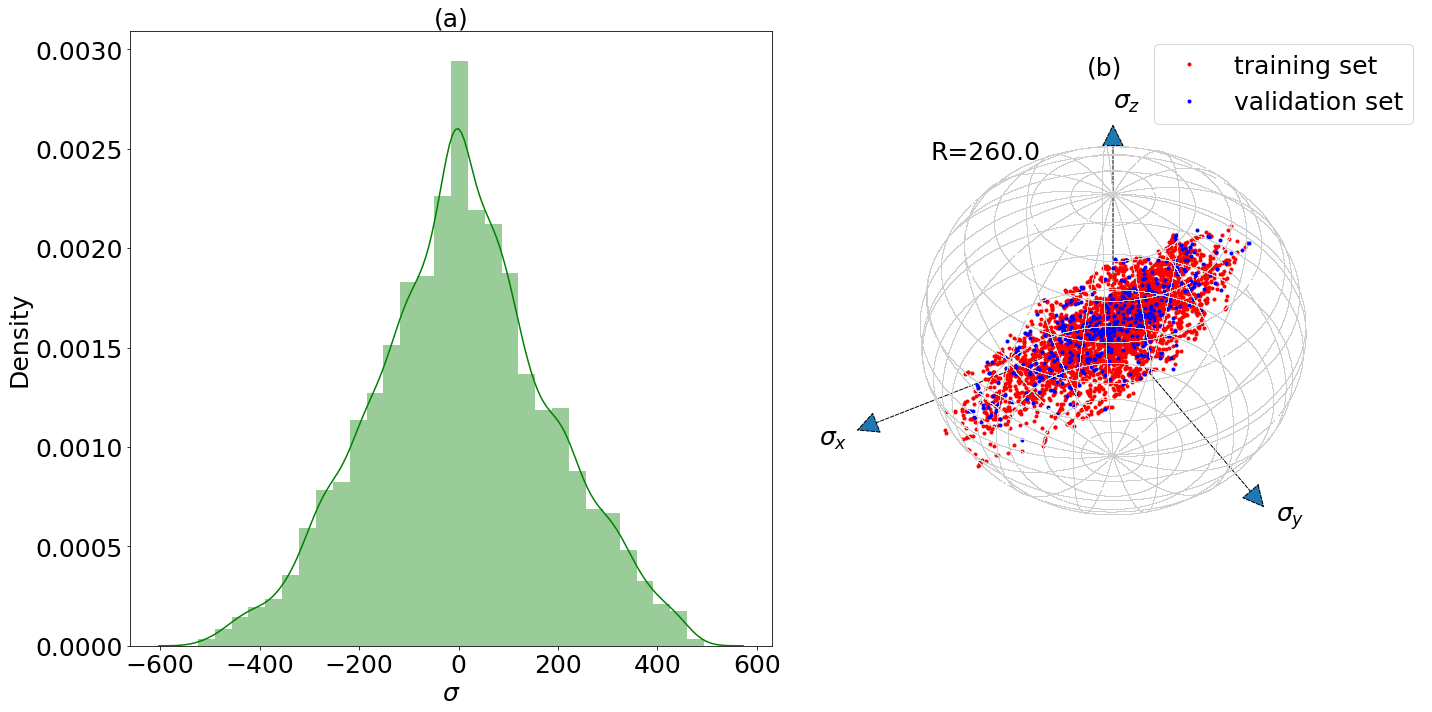


Figure A7. Probability density of the components of $\mathbf{\sigma}$(a, expressed in MPa) and visualisation of the same components in the three Cartesian directions (b), for the case of viscoelastic composites with relaxation expressed by a three-term Prony series.


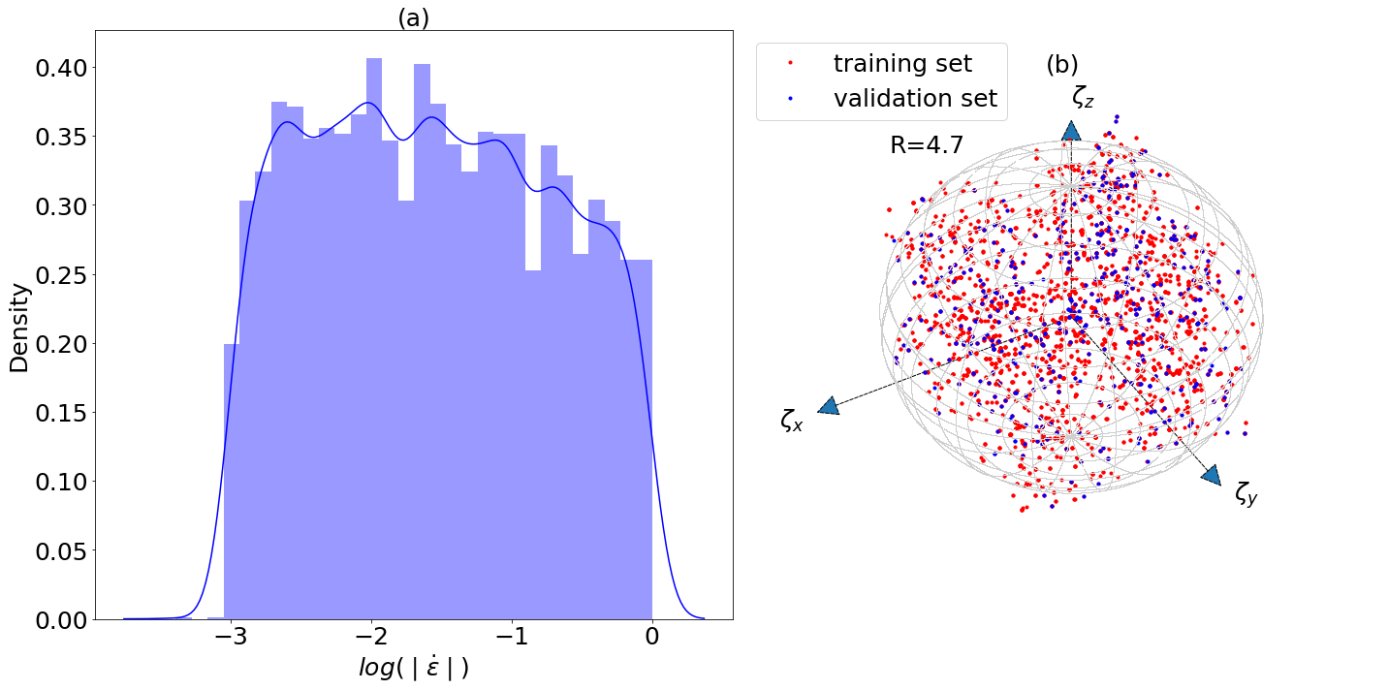


Figure A8. Probability density of the logarithm of the absolute value of the components of (a, with the components having units of s^-1^) and visualisation of the same components in the three Cartesian directions (b), for the case of viscoelastic composites with relaxation expressed by a three-term Prony series. In part (b) we plot , to preserve information about the sign of the strain rate components.


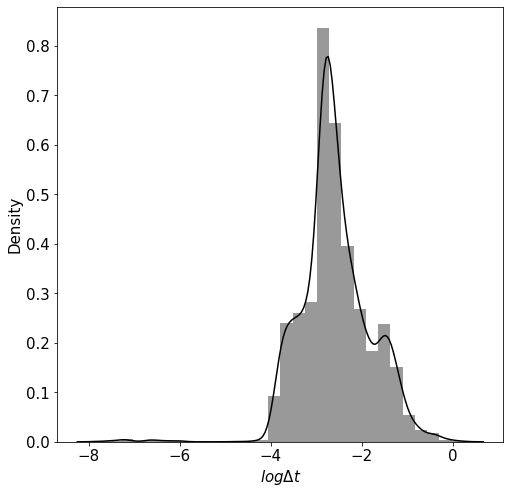


Figure A9. Probability density of $\log\left(\Delta t\right)$ (with $\Delta t$ expressed in seconds) for the case of viscoelastic composites with relaxation expressed by a three-term Prony series.


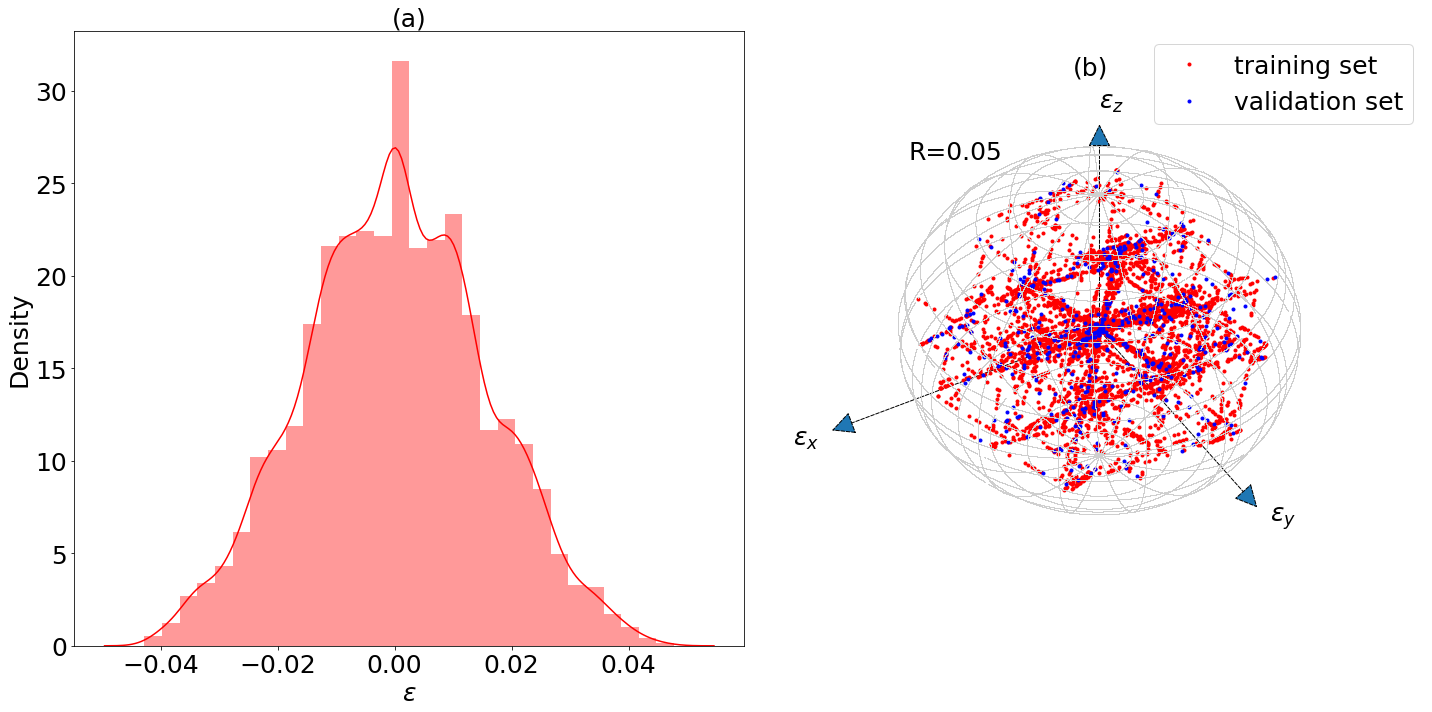


Figure A10. Probability density of the components of $\mathbf{\varepsilon}$(a) and visualisation of the same components in the three Cartesian directions (b), for the case of elastic-plastic composites with uniform yield stress and heterogeneous Young’s modulus.


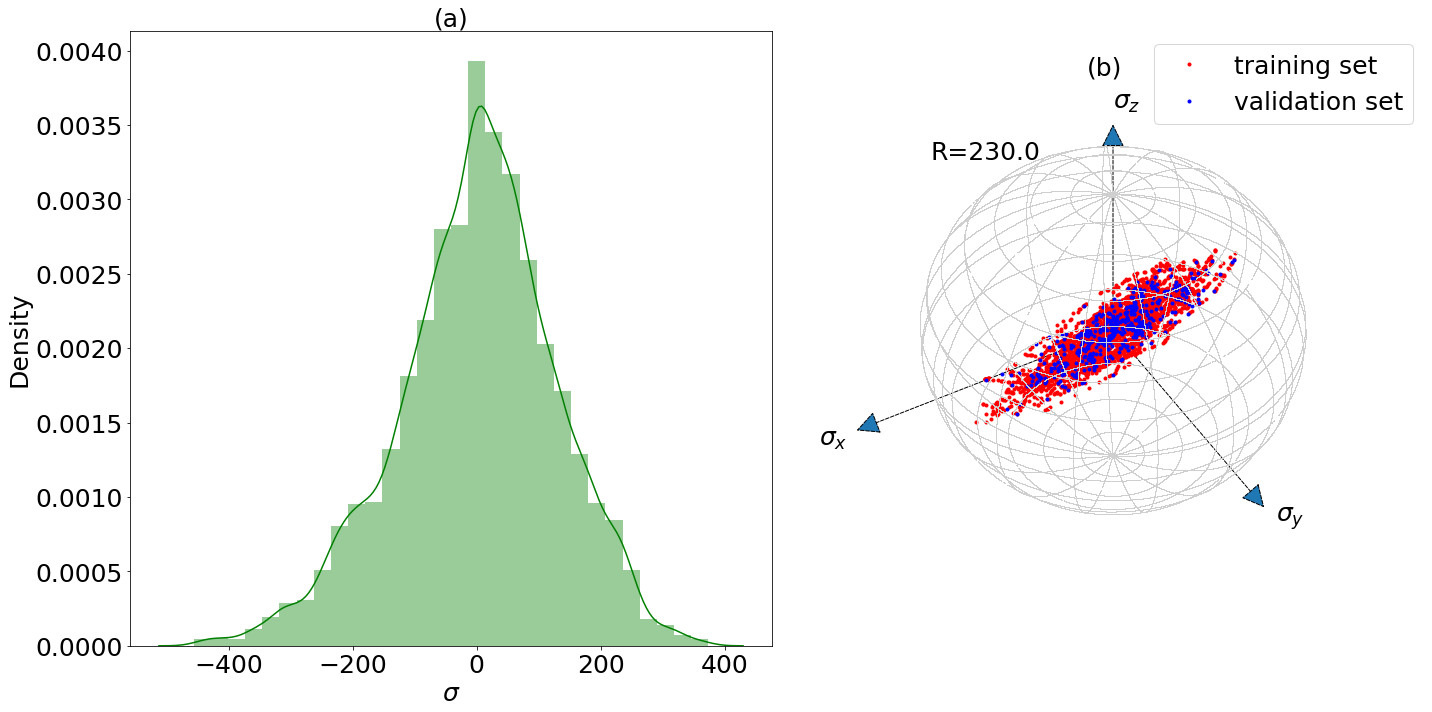


Figure A11. Probability density of the components of $\mathbf{\sigma}$(a, expressed in MPa) and visualisation of the same components in the three Cartesian directions (b), for the case of elastic-plastic composites with uniform yield stress and heterogeneous Young’s modulus.


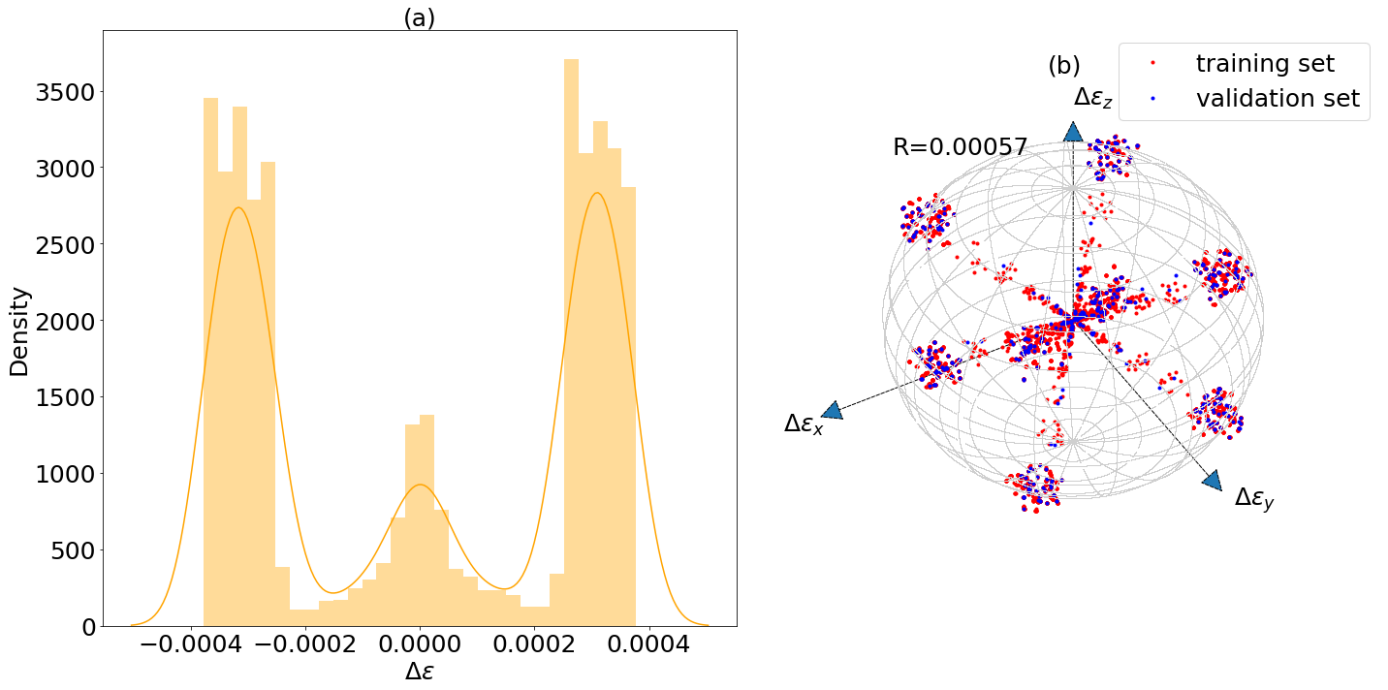


Figure A12. Probability density of the components of $\mathbf{\Delta\varepsilon}$(a) and visualisation of the same components in the three Cartesian directions (b), for the case of elastic-plastic composites with uniform yield stress and heterogeneous Young’s modulus.


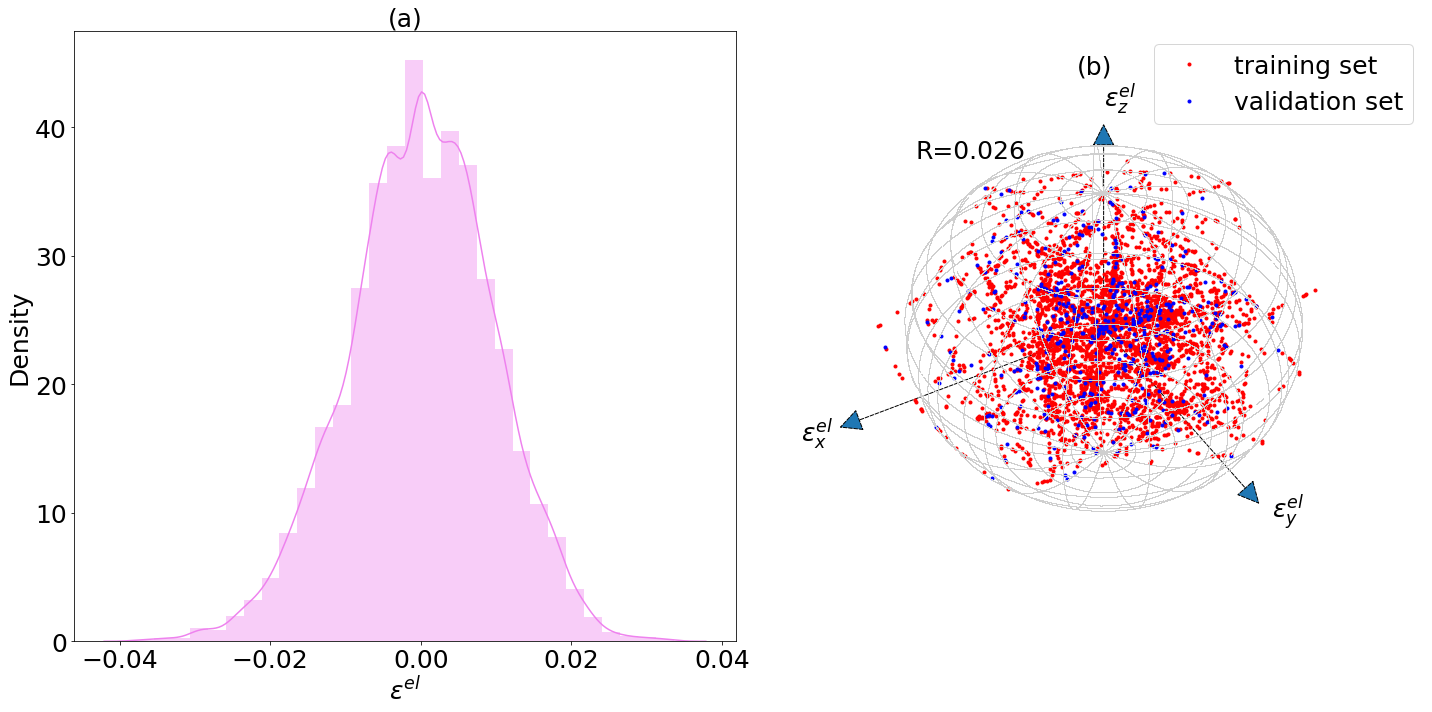


Figure A13. Probability density of the components of $\mathbf{\varepsilon}^{el}$(a) and visualisation of the same components in the three Cartesian directions (b), for the case of elastic-plastic composites with uniform yield stress and heterogeneous Young’s modulus.


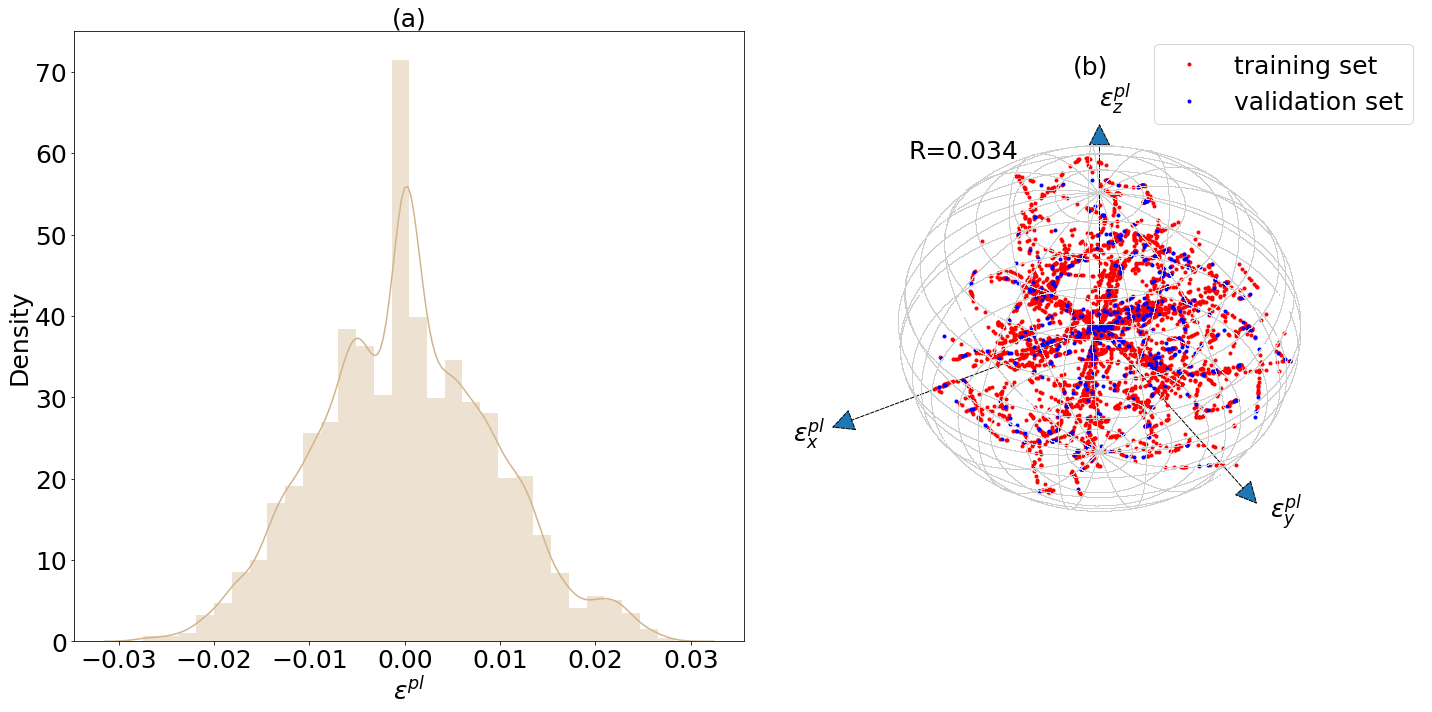


Figure A14. Probability density of the components of $\mathbf{\varepsilon}^{pl}$(a) and visualisation of the same components in the three Cartesian directions (b), for the case of elastic-plastic composites with uniform yield stress and heterogeneous Young’s modulus.


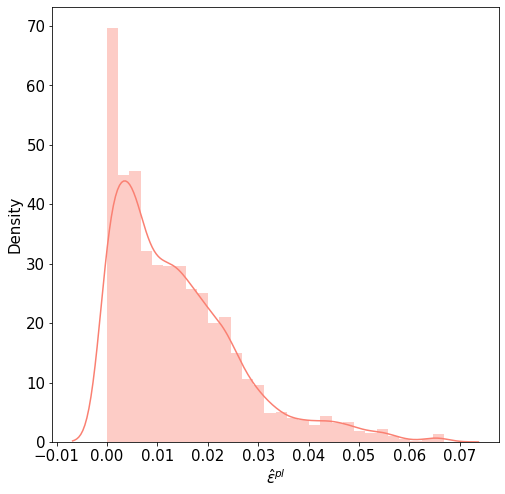


Figure A15. Probability density of $\hat{\varepsilon}^{pl}$for elastic-plastic composites with uniform yield stress and heterogeneous Young’s modulus.


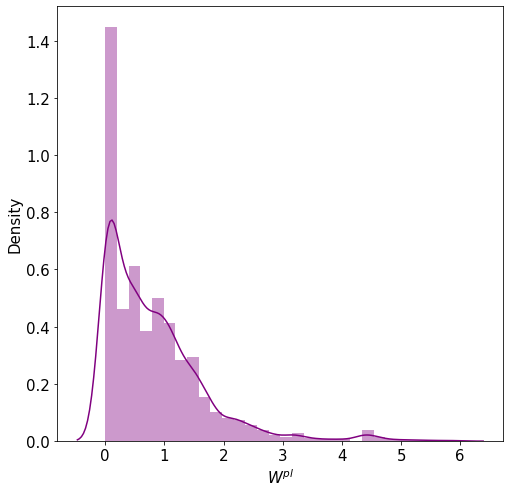


Figure A15. Probability density of $W^{pl}$ (expressed in mJ) for elastic-plastic composites with uniform yield stress and heterogeneous Young’s modulus.


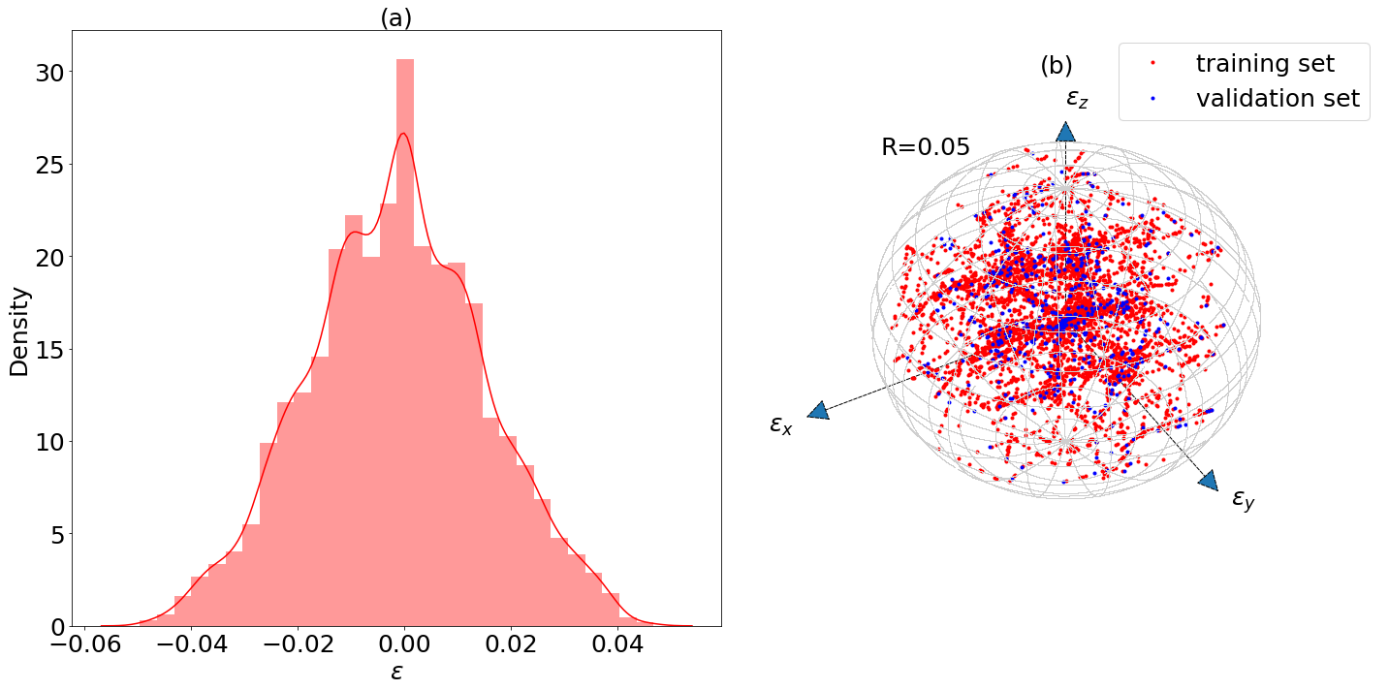


Figure A16. Probability density of the components of $\mathbf{\varepsilon}$(a) and visualisation of the same components in the three Cartesian directions (b), for the case of elastic-plastic composites with uniform Young’s modulus and heterogeneous yield stress.


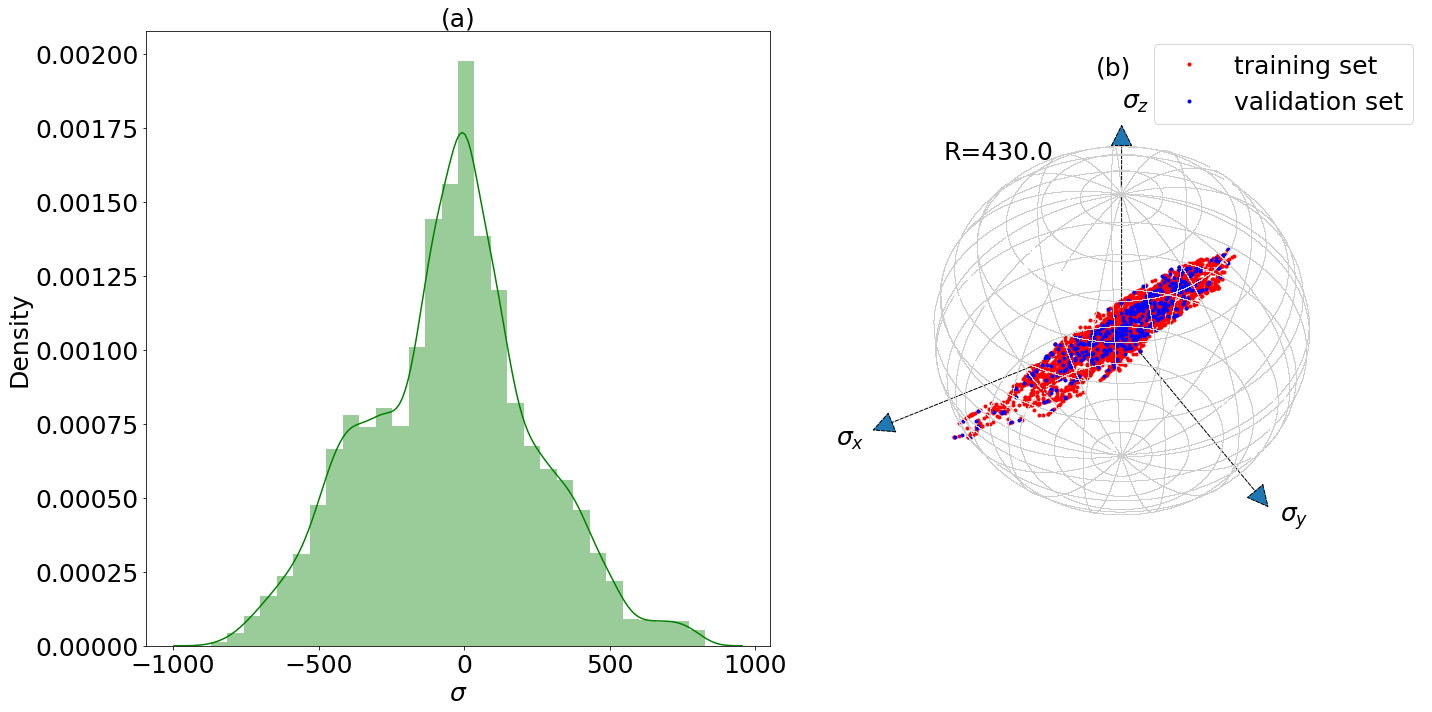


Figure A17. Probability density of the components of $\mathbf{\sigma}$(a, expressed in MPa) and visualisation of the same components in the three Cartesian directions (b), for the case of elastic-plastic composites with uniform Young’s modulus and heterogeneous yield stress.


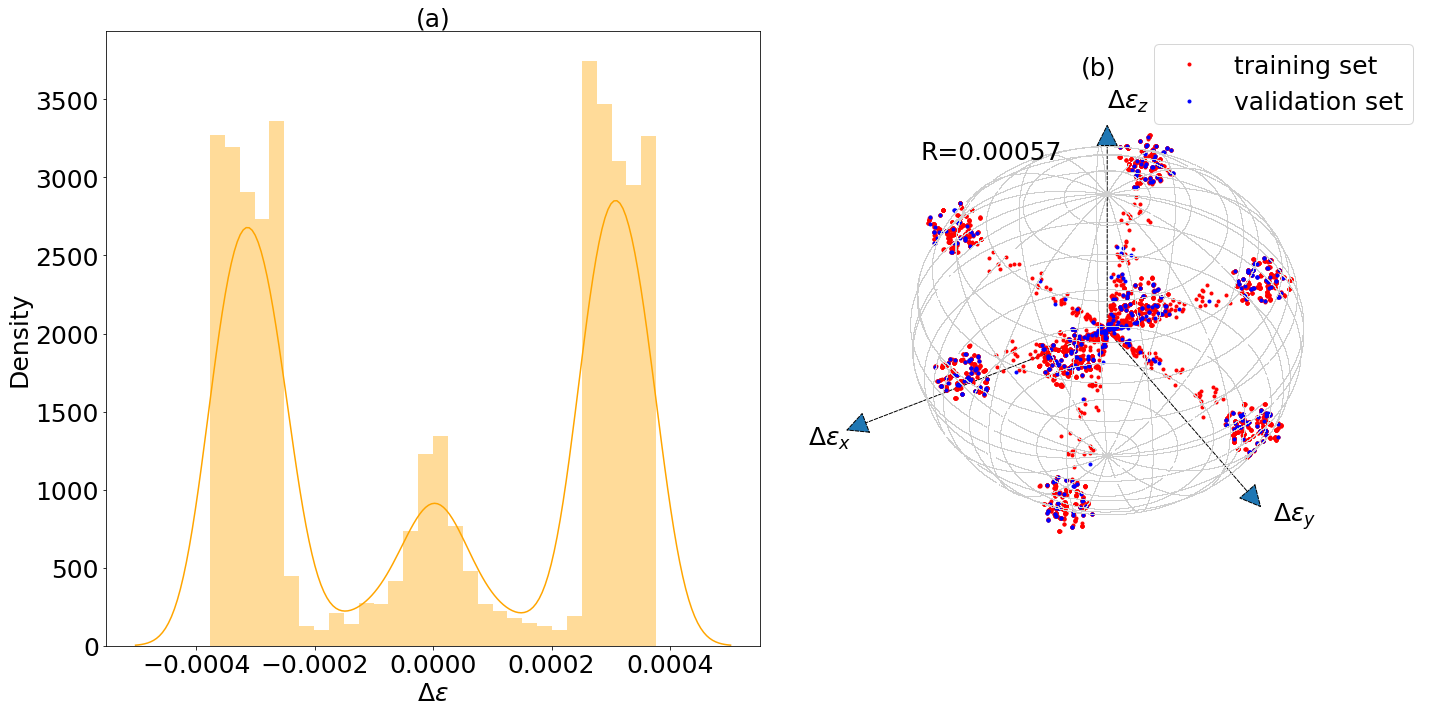


Figure A18. Probability density of the components of $\mathbf{\Delta\varepsilon}$(a) and visualisation of the same components in the three Cartesian directions (b), for the case of elastic-plastic composites with uniform Young’s modulus and heterogeneous yield stress.


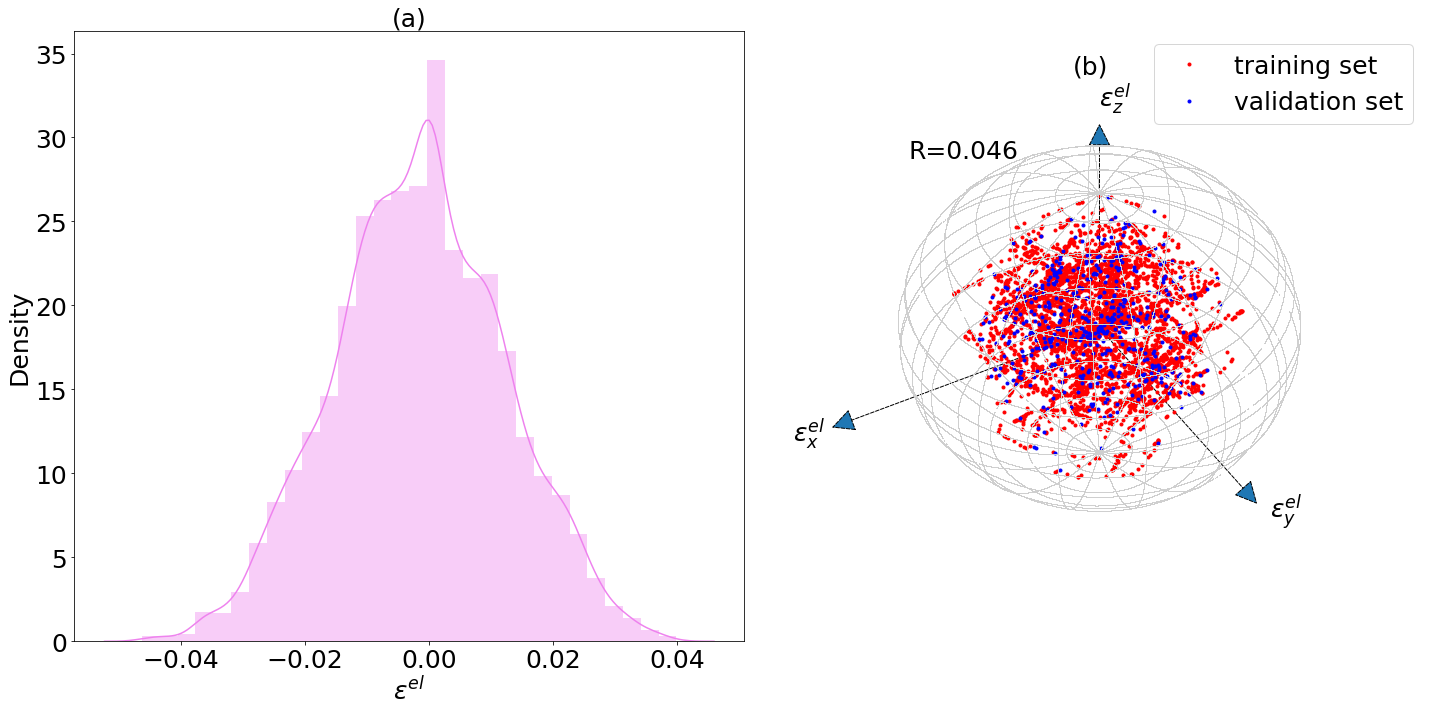


Figure A19. Probability density of the components of $\mathbf{\varepsilon}^{el}$(a) and visualisation of the same components in the three Cartesian directions (b), for the case of elastic-plastic composites with uniform Young’s modulus and heterogeneous yield stress.


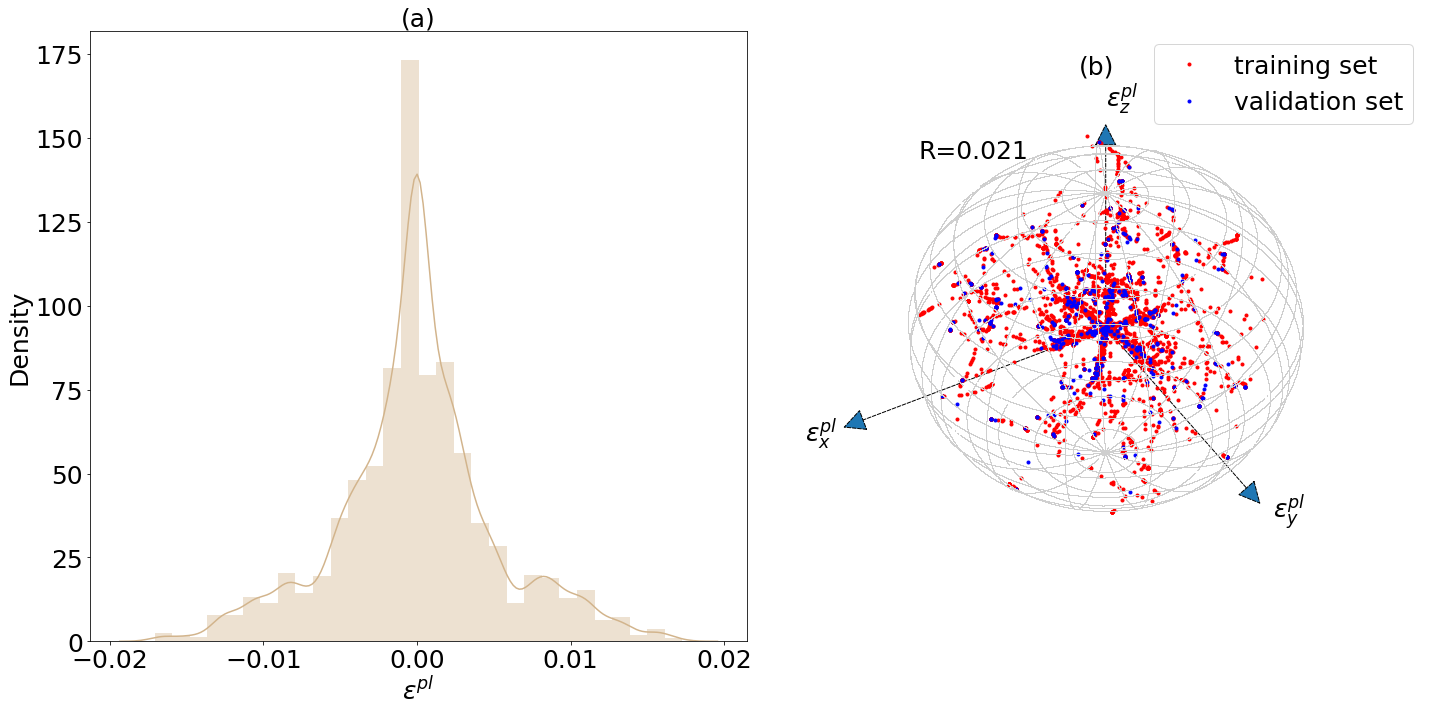


Figure A20. Probability density of the components of $\mathbf{\varepsilon}^{pl}$(a) and visualisation of the same components in the three Cartesian directions (b), for the case of elastic-plastic composites with uniform Young’s modulus and heterogeneous yield stress.


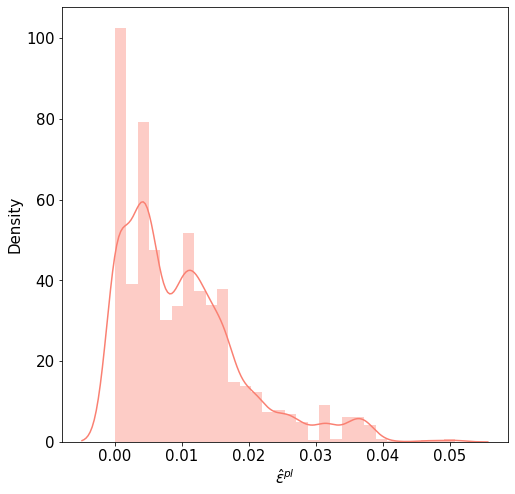


Figure A21. Probability density of $\hat{\varepsilon}^{pl}$for elastic-plastic composites with uniform Young’s modulus and heterogeneous yield stress.


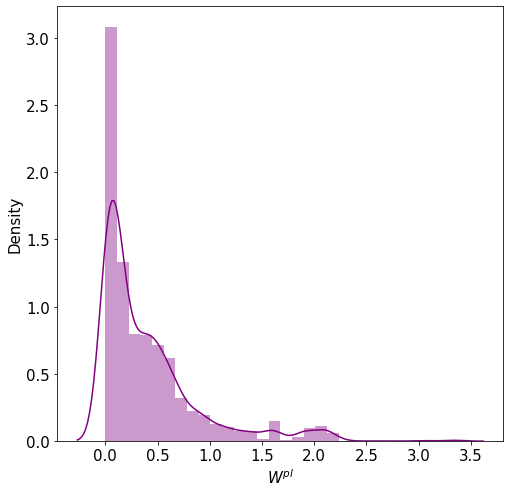


Figure A22. Probability density of $W^{pl}$ (expressed in mJ) for elastic-plastic composites with uniform Young’s modulus and heterogeneous yield stress.

**APPENDIX B: response of a composite with uniform elastic properties and heterogeneous yield stress**

In this Appendix we present information equivalent to that shown in Figs. 9-13, for the case of a composite of uniform stiffness and heterogeneous yield stress. As discussed in Section 3.3, the response of a composite with homogeneous elastic properties is easier to capture than that of a composite with homogeneous plastic properties. Figure B1 and B2 shows that this is indeed the case. A comparison of Figs. B3 and 12 reveals that in this case the classification process is slightly harder than for a composite with uniform plastic properties. In contrast, comparing Figs. B4 and 13 shows that in this case the proposed surrogate models are much more effective in capturing the evolution of state variables over a time increment. The overall result is that the values of $\bar{E}_{\sigma}$ for composites with uniform elastic properties (Fig. B2) are approximately 20% of the corresponding values for composites with uniform plastic properties (Fig. 11).


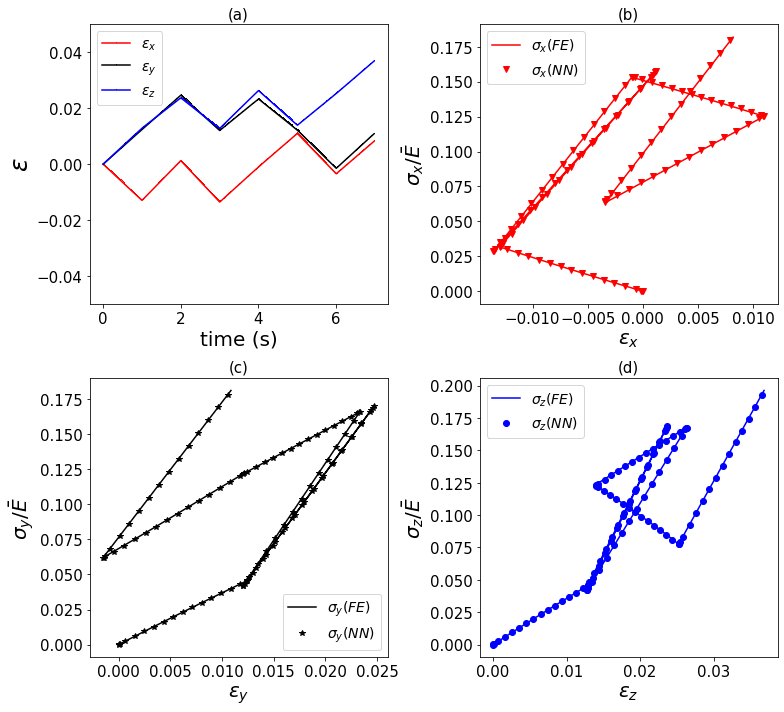


Figure B1. Example of strain versus time (a) and stress versus strain (b,c,d) histories for an elastic-plastic composite with $\kappa=2.0$. FE predictions are compared to predictions by a surrogate model. $E_{\mathbf{\sigma}}=0.3\%$ for the load case shown, which used training datapoints.

Figure B2. Average path-wise stress error $\bar{E}_{\mathbf{\sigma}}$ (calculated over 20 different pseudo-random loading paths) as a function of the heterogeneity $\kappa$ and the number of training datapoints . Data are shown for models I, II and III.


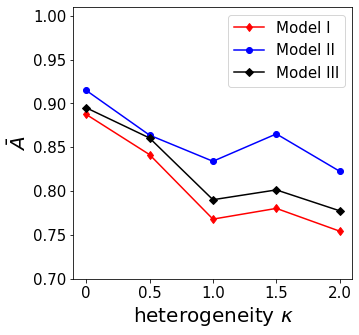


Figure B3. Average path-wise accuracy $\bar{A}$ (calculated over 20 different pseudo-random loading paths) as a function of the heterogeneity $\kappa$. The performance of the classifications in models I, II and III is compared.


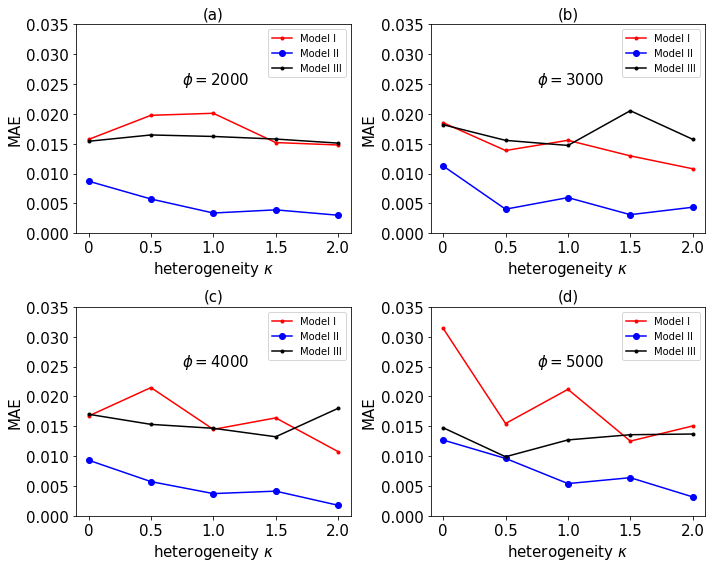


Figure B4. *MAE* for the validation dataset at convergence, for the NNs , and , as a function of the heterogeneity $\kappa$ and the number of training datapoints . Data are shown for models I, II and III.
